# Phenotypic heterogeneity in mortality and prognosis of pulmonary alveolar proteinosis: a large-scale, global pooled analysis of individual-level data

**DOI:** 10.1186/s13023-025-03617-3

**Published:** 2025-03-04

**Authors:** Junfeng Huang, Shuojia Xie, Yuewen Gao, Zikai Lin, Zhe Xu, Jinsheng Lin, Linzhi He, Gengjia Chen, Ziwen Zheng, Zhixing Xu, Jingyan Chen, Jiaming Guo, Zhile Wu, Ailing Duan, Weizhan Luo, Xinyu Song, Shiyue Li

**Affiliations:** 1 Guangzhou Institute of Respiratory Health, State Key Laboratory of Respiratory Disease, National Clinical Research Center for Respiratory Disease, National Center for Respiratory Medicine, The First Affiliated Hospital of Guangzhou Medical University, Guangzhou, Guangdong China; 2 Guangzhou Medical University, Guangzhou, Guangdong China

**Keywords:** Pulmonary alveolar proteinosis, Phenotypic heterogeneity, Prognosis, Genetics

## Abstract

**Background:**

Pulmonary Alveolar Proteinosis (PAP) is a rare interstitial lung disease with diverse clinical manifestations and outcomes. However, there are limited data on the heterogeneity of PAP, as well as its prognosis, cause of death and genetic mechanisms. This study aims to elucidate mortality, prognostic features, and genetic mechanisms in patients with PAP.

**Methods:**

The individual patient data of clinical and mortality were obtained by summarizing the published cases series. Patients with PAP were classified using K-means clustering, and logistic regression identified prognostic factors affecting outcomes. Inheritance and related mechanism of PAP were described by summarizing PAP related genes and enrichment analysis.

**Findings:**

Our analysis included 3278 patients from 295 reports, with 88.6% diagnosed with idiopathic PAP (IPAP). Twelve major categories of cause were counted from 312 deaths (mortality: 9.5%), the most common of which were respiratory failure (45.8%) and lung infections (18.3%). Three symptom-related clusters were identified, and patients with multiple symptoms appeared to have worse mortality than those with single or no symptoms (*p* < 0.05). Non-secondary patterns (OR 2.87, *p* = 0.003), whole lung lavage (OR 0.15, *p* < 0.001), and effective GM-CSF therapy (OR 0.08, *p* < 0.001) are prognostic factors associated with decreased mortality. Additionally, 134 significant genes related to PAP development were identified, highlighting the roles of immune response and lipid metabolism.

**Interpretation:**

This study comprehensively describes the clinical characteristics cause of death, prognosis and associated factors based on the global PAP population. The significant phenotype heterogeneity highlighting the importance of long-term prognosis and individualized management for patients with PAP.

**Supplementary Information:**

The online version contains supplementary material available at 10.1186/s13023-025-03617-3.

## Introduction

Pulmonary Alveolar Proteinosis (PAP) is a rare respiratory disease characterized primarily by abnormal deposition of surfactant in the alveoli [1]. Based on the etiology, it can be categorized into primary PAP, secondary PAP, and congenital PAP. Primary PAP is further divided into autoimmune and genetic forms, with autoimmune PAP (APAP) being the most prevalent, accounting for over 85% of cases [2]. Globally, the prevalence of PAP is estimated at 7.0 per one million, with APAP accounting for over 85% [3]. The clinical spectrum of PAP ranges from spontaneous remission and stability to severe outcomes, including death [4]. However, prior epidemiological investigations on PAP were limited to clinical observations, constraining further understanding in the evolution of outcomes and individualized management [5, 6]. Moreover, the causes of death and prognosis associated with different PAP patterns remain largely unexplored.

Clinically, PAP presents with nonspecific respiratory symptoms such as dyspnea and cough. [6] High-resolution CT scans typically show a paver-like pattern [7]. However, limited studies have identified significant heterogeneity in the clinical phenotype and outcomes of PAP. Patients may exhibit a range of symptoms from severe to mild or even asymptomatic, despite having characteristic radiographic findings [8–10]. Interestingly, some patients with severe symptoms may maintain normal pulmonary function levels [8], while asymptomatic patients may face poorer prognoses [11–13]. This highlights the need for systematic research into the impact of phenotypic heterogeneity on clinical outcomes in PAP. Whole lung lavage (WLL) and granulocyte–macrophage colony-stimulating factor (GM-CSF) therapy are established treatment options, with clinical improvement often used as a marker of efficacy [1]. However, evidence suggests that these treatments may not always benefit patients [14, 15], with ongoing research needed to fully understand their impact on clinical outcomes and long-term prognosis. Concurrently, current research mainly focuses on short-term benefits, with a lack of exploration into prognostic outcomes like mortality in PAP patients.

The primary pathogenesis of PAP involves the aberrant deposition of pulmonary surfactant within alveolar macrophages and spaces [1]. Recent studies hint at genetic regulation role in progression and clinical variability of PAP, though genetic data remain unclear [2]. In this context, this study aims to summarize global PAP clinical features, describe prognosis comprehensively, explore factors affecting prognosis, and identify phenotypic characteristics linked to prognosis, while further elucidating related genetic traits and mechanisms.

## Method

### Study design

**Fig. 1:**
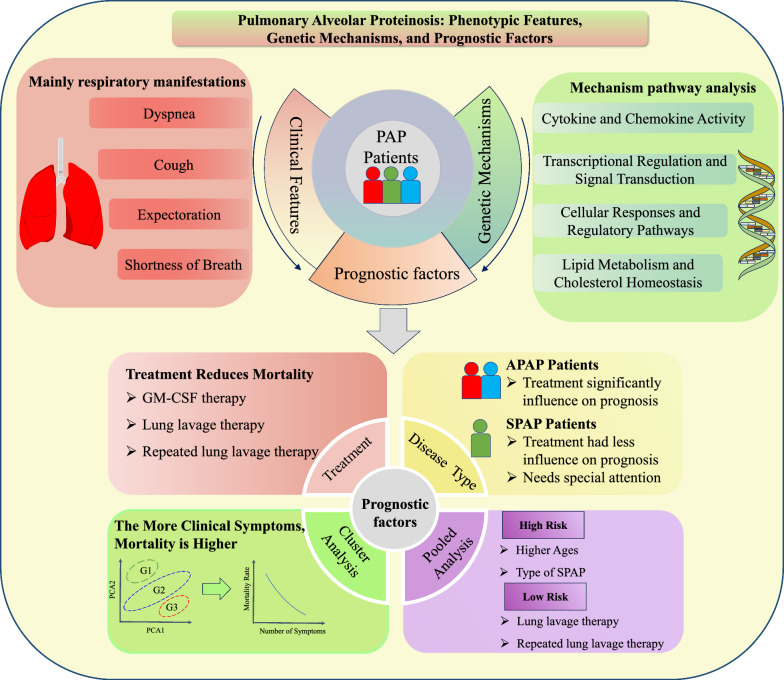
Graphical abstract of the exploratory study based on clinical characteristics, genes and prognostic factors of the Global PAP Pooled Analysis. *GM-CSF* granulocyte–macrophage colony-stimulating factor; *PAP* pulmonary alveolar proteinosis; *SPAP* secondary pulmonary alveolar proteinosis

The flowchart of our study is shown in Fig. 1. Clinically, we aggregated the clinical characteristics of the global population with PAP and integrated this with the contents of rare disease and disease phenotype databases to provide a comprehensive description of the clinical features of PAP. In exploring factors related to the prognosis and mortality of PAP, clinical phenotypes, treatment interventions, and the variations among different types of PAP were examined. This was further complemented by individualized exploration in small samples to comprehensively elucidate the impact of these factors on the mortality outcomes of PAP. Genes and mechanistic pathways were analyzed through the collation of research materials and the incorporation of information from PAP-related gene databases. Specific methods and descriptions can be found in the Supplement Material.

### Data analysis

The quantitative data were presented as mean ± SD or median (interquartile range 25–75%) according to distribution. Comparisons were analyzed with the T-test for normally distributed data, or the Wilcoxon signed-rank test for non-normal data. The chi-square test was used to examine categorical variables. K-means clustering was used to explore differences in mortality and other clinical characteristics across corresponding clinical phenotypes. Follow-up patients with a balanced follow-up time were included in the survival comparative analysis to ensure comparability and accuracy. Logistic regression analysis was conducted to determine the relative risk associated with mortality of PAP patients in relation to clinical information and intervention measures. Gene Ontology (GO) enrichment analysis was performed using the DAVID platform (DAVID, https://david.ncifcrf.gov/). Pathways with FDR < 0.05 were considered significant in enrichment analysis. Differences with a *p* < 0.05 were considered statistically significant in other analysis. All statistical analyses were performed in R (version 4.2.0).

### Patient and public involvement

Patients and the public were not be involved.

## Results

### Basic characteristics of the study

**Fig. 2:**
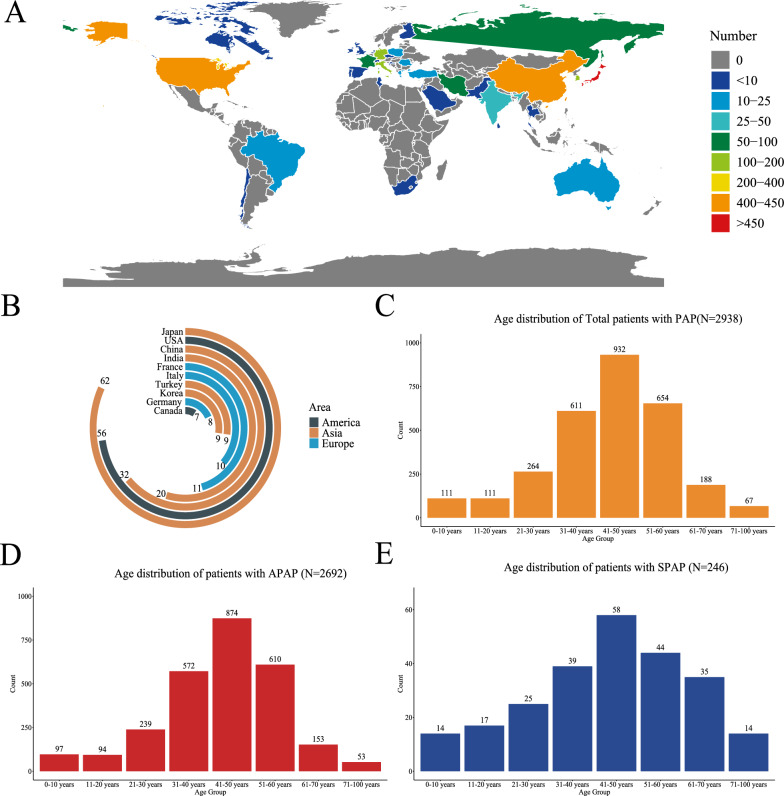
Global PAP Cohort population characteristics. **A** Global distribution diagram of Global PAP Cohort Analysis; **B** The top ten countries included in the Global PAP Cohort Analysis; **C** Age distribution of Total PAP patients (N = 2938); **D** Age distribution of APAP patients (N = 2692); **E** Age distribution of SPAP patients (N = 246)

In total, 295 studies including 3278 patients with PAP met the inclusion criteria (Supplementary Table A1). Most of the studies (96.3%) were performed in the twenty-first century, with nearly half in Asia (49.5%) (Table 1). Supplementary Fig. 2B shows that the largest number of studies were in Japan, followed by the US and China. The global distribution of PAP patients is shown in Supplementary Fig. 2A, the largest number of patients came from Japan, with 1195 PAP patients (36.46%), followed by the United States (13.27%) and China (12.72%). Most cases were in the 41–50-year age group (N = 932) (Supplementary Fig. 2C). The age distributions were similar in both types of PAP (Supplementary Fig. 2D/Fig. 2E).

**Table 1:** Basic information on studies included in the Global PAP Pooled Analysis

Variable	Study (n)
Total (n)	295
Patients (n)	
N > 50	13
N = 50–10	40
N < 10	242
Area	
Asia	146
Europe	64
America	68
Oceania	6
Others	11
Year	
2011–2021	179
2001–2010	105
—2000	11

### Clinical characteristics

Table 2 details the clinical characteristics of PAP patients. Diagnosis was primarily through bronchoscopy (N = 1322) or lung biopsy (N = 865), with GM-CSF autoantibody status aiding further classification (N = 1182). Patients were categorized into three types, with IPAP being the most prevalent (N = 2903). Less than 30% of patients received WLL (N = 969), with only 409 (12.5%) receiving multiple WLL. The main clinical manifestations were respiratory [e.g., dyspnea (N = 1616) and cough (N = 1167)]. Data from Orphanet and Human Phenotype Ontology databases align with our findings, showing that respiratory symptoms are predominant in patients (Supplementary Table A2/Table A3). For IPAP patients (N = 153), the main complications were pulmonary infections (N = 101), followed by respiratory disease (N = 16) and endocrine disorders (N = 12) (Supplementary Fig. 3A). Pulmonary infections were mostly bacterial, with Nocardia being the most common pathogen (N = 48) (Supplementary Fig. 3B). In the SPAP cohort (N = 288), hematological disease was the leading cause (N = 134), with myelodysplastic syndrome being the most frequent (N = 56) (Supplementary Fig. 3C).

**Table 2:** Clinical and prognostic characteristics for 3278 global PAP population

Variable	Total	IPAP	SPAP	CPAP
Patients (n)	3278	2903	288	87
Age (years)	43	41	51	5
Diagnostic approach (%)				
Bronchoscopic biopsy	1322 (40.3)	1204 (41.5)	80 (27.8)	38 (43.7)
Lung biopsy	865 (26.4)	730 (25.1)	100 (34.7)	35 (40.2)
GM-CSF antibodies	1182 (36.1)	1020 (35.1)	143 (49.7)	19 (21.8)
Autopsy	46 (1.4)	23 (0.8)	22 (7.6)	1 (1.1)
Clinical manifestations (%)				
Dyspnea	1616 (49.3)	1442 (49.7)	153 (53.1)	21 (24.1)
Cough	1167 (35.6)	1036 (35.7)	116 (40.3)	15 (17.2)
Expectoration	261 (8.0)	251 (8.6)	8 (2.8)	2 (2.3)
Shortness of Breath	88 (2.7)	56 (1.9)	18 (6.3)	14 (16.1)
Fever	510 (15.6)	404 (13.9)	100 (34.7)	6 (6.9)
Weight Loss	459 (14.0)	443 (15.3)	11 (3.8)	5 (5.7)
Fatigue	443 (13.5)	440 (15.2)	2 (0.7)	1 (1.1)
Others	130 (4.0)	71 (2.4)	30 (13.5)	20 (23.0)
Treatment (%)				
Lung lavage	969 (29.6)	838 (28.9)	73 (25.3)	61 (70.1)
Repeated lung lavage	409 (12.5)	361 (12.4)	34 (11.8)	14 (16.1)
GM-CSF	312 (9.5)	286 (9.9)	22 (7.6)	4 (4.6)
Outcome (%)				
Spontaneous remission	85 (2.6)	79 (2.7)	6 (2.1)	0 (0.0)
Stabilization	2881 (87.9)	2625 (90.4)	204 (70.8)	53 (60.9)
Deaths	312 (9.5)	196 (6.8)	81 (28.1)	35 (40.2)

*PAP* pulmonary alveolar proteinosis; *IPAP* idiopathic pulmonary alveolar proteinosis; *SPAP* secondary pulmonary alveolar proteinosis; *CPAP* congenital pulmonary alveolar proteinosis; *GM-CSF* granulocyte–macrophage colony-stimulating factor

**Table 3:** Clinical outcome and cause of death of global PAP population

Variable	Total	PAP Classification		Area				Publication year		
		Non-SPAP	SPAP	Asia	America	Europe	Other Area	≤ 2000	≤ 2010	> 2010
Study (n)	295	168	127	145	68	64	18	11	105	179
Patients (n%)	3278	2990	288	1886	459	515	418	69	1341	1868
Death, n (%)^1^	312 (9.5)	231 (7.7)	81 (28.1)	125 (6.6)	50 (10.9)	66 (12.8)	71 (17.0)	27 (39.1)	111 (8.3)	174 (9.3)
Cause of Death, n (%)^2^										
Respiratory failure	143 (45.8)	108 (34.6)	35 (43.2)	36 (28.8)	16 (32.0)	42 (63.6)	49 (69.0)	3 (11.1)	61 (55.0)	79 (45.4)
Lung infections	57 (18.3)	36 (11.5)	21 (25.9)	29 (23.2)	15 (30.0)	5 (7.6)	8 (11.3)	14 (51.9)	16 (14.4)	27 (15.5)
Hematological disease	15 (4.8)	4 (1.3)	11 (13.6)	12 (9.6)	3 (6.0)	0 (0.0)	0 (0.0)	2 (7.4)	1 (0.9)	12 (6.9)
Cancer	12 (3.8)	11 (3.5)	1 (1.2)	6 (4.8)	4 (8.0)	0 (0.0)	2 (2.8)	2 (7.4)	5 (4.5)	5 (2.9)
Complication of WLL	7 (2.2)	7 (2.2)	0 (0.0)	4 (3.2)	2 (4.0)	0 (0.0)	1 (1.4)	1 (3.7)	3 (2.7)	3 (1.7)
Brain abscess	7 (2.2)	7 (2.2)	0 (0.0)	0 (0.0)	2 (4.0)	1 (1.5)	4 (5.6)	2 (7.4)	4 (3.6)	1 (0.6)
Sepsis	6 (1.9)	1 (0.3)	5 (6.2)	2 (1.6)	2 (4.0)	2 (3.0)	0 (0.0)	0 (0.0)	3 (2.7)	3 (1.7)
Digestive disease	3 (1.0)	3 (1.0)	0 (0.0)	1 (0.8)	1 (2.0)	0 (0.0)	1 (1.4)	1 (3.7)	1 (0.9)	1 (0.6)
Cardiovascular disease	5 (1.6)	3 (1.0)	2 (2.5)	0 (0.0)	1 (2.0)	3 (4.5)	1 (1.4)	0 (0.0)	3 (2.7)	2 (1.1)
Lung transplantation	3 (1.0)	2 (0.6)	1 (1.2)	0 (0.0)	0 (0.0)	3 (4.5)	0 (0.0)	0 (0.0)	1 (0.9)	2 (1.1)
Other	10 (3.2)	7 (2.2)	3 (3.7)	1 (0.8)	2 (4.0)	6 (9.1)	1 (1.4)	2 (7.4)	3 (2.7)	5 (2.9)
Unknown	44 (14.1)	42 (13.5)	2 (2.5)	34 (27.2)	2 (4.0)	4 (6.1)	4 (5.6)	0 (0.0)	10 (9.0)	34 (19.5)

^1^Ratio represents the ratio of deaths to the number of people in a group

^2^Ratio represents the ratio of deaths to the number of deaths within a group

*PAP* pulmonary alveolar proteinosis; *SPAP* secondary pulmonary alveolar proteinosis; *ALL* acute lymphoblastic leukemia; *AML* acute myeloid leukemia; *MDS* myelodysplastic syndromes; *WLL* whole lung lavage

### Exploration of prognostic factors

#### Summary of prognosis

A total of 2696 patients were followed for five years, and the mean follow-up time of patients was 1.82 ± 1.60. Over 85% of the patients had stable clinical symptoms, with 312 (9.5%) dying during follow-up and 85 (2.6%) experiencing spontaneous remission of symptoms (Table 2). Mortality rates and causes are detailed in Table 3. The overall mortality rate was 9.5%, with a notable difference between SPAP (28.1%) and non-SPAP groups (7.7%) (*p* < 0.05). Geographically, the lowest mortality rate was in Asia (6.6%), and pre-2000 studies reported a high mortality rate of 39.1%. Major causes of death included respiratory failure (143/312) and respiratory tract infections (57/312), with Nocardia accounting for 4.8% of deaths. Other causes of death included various bacterial infections (1.3%), cryptococcus (1.3%), and so on. Respiratory failure remained the principal cause of death in the PAP subgroups. Hematological diseases, particularly acute myeloid leukemia (7.4%) and myelodysplastic syndrome (4.9%), were a common cause of death in patients with SPAP.

#### Prognostic phenotype

During the follow-up period, 567 patients with asymptomatic were reported to be alive, and 77 patients were dead. Among the patients who simultaneously exhibited symptoms of cough, shortness of breath, and dyspnea, 5 were alive, and 8 had died. Clustering analysis of the incidence rates of cough, shortness of breath, and dyspnea identified six distinct clusters; one with low incidence rates for all three symptoms (G1:C1), three with predominantly one of the three symptoms (G2:C2 + C3 + C5), and two with two symptoms (G3:C1 + C6) (Supplementary Figure A2). The mortality rate was significantly lower in the subgroups in G1 (7.16%) or G2 (8.61%) than in G3 (13.81%) (*p* < 0.05) (Table 4 and Supplementary Figure A3). Mortality attributable to respiratory disease was significantly higher in the G2 (72.34%) and G3 (72.92%) than in the G1 (50.41%). Respiratory failure accounted for a significant proportion of deaths (> 60%) caused by respiratory problems across these three groups. There was a significant difference in the incidence of respiratory failure as a cause of death between G1 (62.30%) and G3 (79.05%) (*p* < 0.001). The proportions of men were significantly lower in G1 (48.73%) and G2 (59.71%) than in G3 (69.61%) (*p* < 0.05). IPAP was the predominant type of PAP in all three groups, particularly G1 (90.17%) and G3 (89.65%). Lavage was performed significantly less often in G1 than in G2 or G3 (*p* < 0.05).

**Table 4:** Clinical characteristics and outcomes across 3 PAP cluster groups

Characteristic	Group			*P* value		
	G1 (N = 1689)	G2 (N = 546)	G3 (N = 1043)	G1 vs G2	G1 vs G3	G2 vs G3
Number of Death (%)	121 (7.16)	47 (8.61)	144 (13.81)	0.308	< 0.001	0.003
Number of deaths attributed to the Respiratory System (%)	61 (50.41)	34 (72.34)	105 (72.92)	0.016	< 0.001	> 0.999
Number of deaths attributed to Respiratory failure (%)	38 (62.30)	22 (64.71)	83 (79.05)	0.091	< 0.001	0.260
Number of deaths attributed to Lung Infections (%)	23 (47.70)	12 (35.29)	22 (20.95)	0.470	0.521	0.169
Sex Male (%)	823 (48.73)	326 (59.71)	726 (69.61)	0.524	0.029	0.018
PAP Type: Primary (%)	1523 (90.17)	445 (81.50)	935 (89.65)	< 0.001	0.704	< 0.001
Frequency of alveolar lavage	0.45 (0.45)	0.62 (0.46)	0.64 (0.45)	0.008	0.006	0.910
Frequency of repeated lung lavage	0.25 (0.38)	0.40 (0.47)	0.34 (0.45)	0.104	0.367	0.414

1. G1, asymptomatic group; G2, single symptom group; G3, two symptoms group

2. T-test or wilcoxon signed-rank test was used for continuous variables; Chi-square test was used for categorical variables

In G2 subgroups (Supplementary Table A4 and Supplementary Figure A3), the mortality rate was higher with cough (C2) than with dyspnea (C5) (19.3% vs. 6.4%, *p* < 0.001). Most deaths in G2 were due to respiratory complications, with similar rates across C2 (70.59%), C3 (100%), and C5 (71.43%). IPAP prevalence was lower in C3 (59.09%) compared to C5 (83.26%) (*p* < 0.001). Lavage rates were lower in C2 than in C3 and C5 (*p* < 0.05).

In G3 subgroups (Supplementary Table A5), mortality was higher with cough and dyspnea (C6) compared to cough and shortness of breath (C1) (*p* < 0.05). There was no significant difference in sex distribution or mortality due to specific respiratory diseases. IPAP was more common in C6 (91.54%) than C1 (15.38%) (*p* < 0.05). Deaths attributed to respiratory disease were higher in C6 (75.57%) compared to C1 (46.16%) (*p* < 0.05).

### Therapeutic interventions and subtypes

In patients for whom the three therapeutic interventions and outcomes were clearly defined, the mortality rates were 4.9% for GM-CSF, 3.5% for single-session (initial) whole lung lavage (WLL), and 2.5% for repeated WLL. These rates were significantly lower compared to the overall population (*p* < 0.05) (Supplementary Fig. 4A). Furthermore, mortality was notably lower in patients who responded to GM-CSF (0.5%) compared to non-responders (9.4%) (Supplementary Figure A4). In the non-SPAP population, treatment significantly reduced mortality (Supplementary Fig. 4B). For SPAP patients, WLL and repeated WLL showed a more pronounced effect on mortality (Supplementary Fig. 4C). Patients with a hematological disorder (72.7%) as the primary secondary factor had the highest mortality rate, which was significantly higher than in the overall SPAP population (28.1%) (*p* < 0.05) (Supplementary Fig. 4D). In addition, correlation analyses showed that increased WLL and repeated WLL frequencies were significantly associated with lower long-term mortality. The presence of crazy-paving patterns on CT was not significantly associated with long-term mortality in PAP patients. (Supplementary Figure A5).

### Individualized exploration of PAP prognostic factors

Supplementary Table A6 shows the inclusion criteria for the individual PAP cohort, in which 41 of 211 patients (19.4%) died within a 5-year period. The age and sex distributions of these 211 patients are shown in Supplementary Figure A6. Differences in age distribution are discernible in Supplementary Figure A7, where the age of the patients who died in the total population was significantly higher than that of those who survived (*p* < 0.05). There was no significant age difference between the sexes or between SPAP and the other PAP subtypes. Subgroup analysis revealed significant age differences in the male and SPAP subgroups (Supplementary Figure A8). Furthermore, there were significant differences between the deceased and surviving groups in the total population according to whether WLL was received and type of PAP (Supplementary Table A7). Logistic regression identified higher death risk for patients over 50 years old (OR 2.30, 95% CI 1.12–4.70, *p* = 0.022) and those with SPAP (OR 2.87, 95% CI 1.43–5.84, *p* = 0.003) (Supplementary Fig. 5A). Conversely, WLL (OR 0.15, 95% CI 0.07–0.32, *p* < 0.001), repeated WLL (OR 0.32, 95% CI 0.14–0.66, *p* = 0.003), and effective GM-CSF therapy (OR 0.08, 95% CI 0.01–0.44, *p* < 0.001) were associated with lower death risks. Lower death risk was also noted in patients receiving alveolar lavage (OR < 1, *p* < 0.05) (Supplementary Fig. 5B/C).

### Gene mechanism analysis

**Fig. 3:**
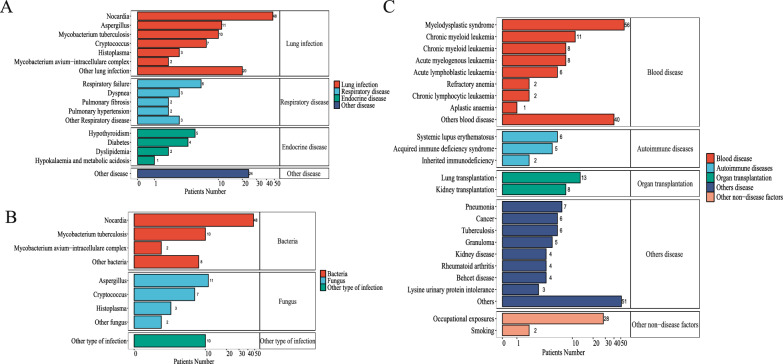
Clinical Characteristics of PAP: Infection Spectrum, IPAP Complications, and SPAP Etiologies. **A** Complications of IPAP; **B** Bacterial spectrum of pulmonary infection in IPAP patients; **C** Secondary factors of SPAP

**Fig. 4:**
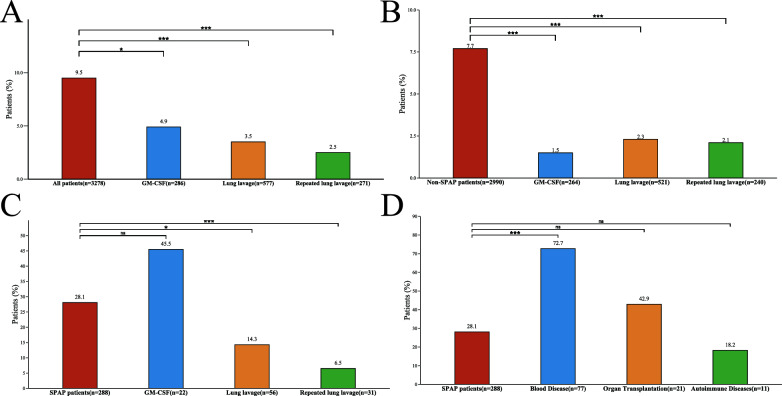
Effect of PAP treatment intervention on mortality. **A** Effect of treatment on mortality in total PAP population; **B** Effect of treatment on mortality in Non-SPAP population; **C** Effect of treatment on mortality in SPAP population; **D** Effects of major secondary factors on mortality in SPAP. *GM-CSF* granulocyte–macrophage colony-stimulating factor; *PAP* pulmonary alveolar proteinosis; *SPAP* secondary pulmonary alveolar proteinosis

**Fig. 5:**
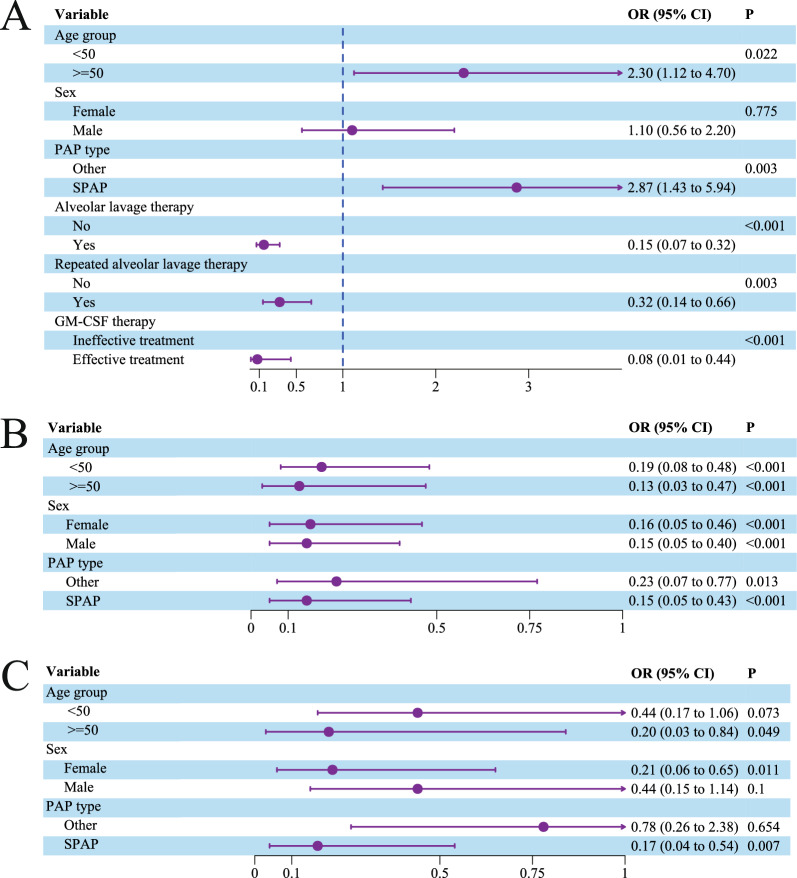
Association of clinical characteristics and treatment with outcomes in 211 patients with PAP in pooled analysis. **A** Effect of clinical characteristics and treatment on death outcomes in 211 PAP patients; **B** Effect of lung lavage on death outcomes in different subgroups; **C** Effect of repeated lung lavage on death outcomes in different subgroups. *GM-CSF* granulocyte–macrophage colony-stimulating factor; *PAP* pulmonary alveolar proteinosis; *SPAP* secondary pulmonary alveolar proteinosis

**Fig. 6:**
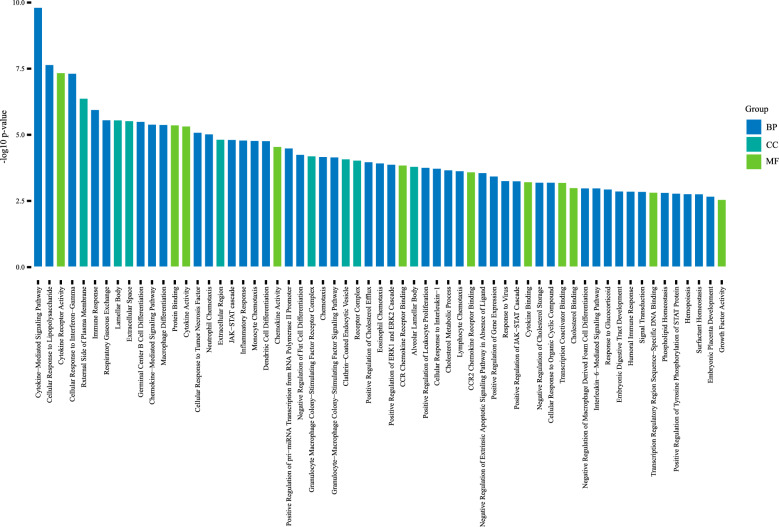
GO enrichment analysis result of the top 77 PAP related genes based on GeneCards database. *BP* biological process; *MF* molecular function; *CC* cellular component

From the global 3278 PAP population study, a variety of gene mutations were identified across different years, with some of the most notable including GATA-2 and CSF2RA (Supplementary Table A8). On this basis, we retrieved the genes and gene scores related to PAP (Supplementary Table A9) and its three types (Supplementary Table A10/11/12) from the GeneCards database. A total of 134 PAP-related genes were identified, with 85% highly relevant genes being protein-coding, including MARS1 (Figs. 2, 3, 4, 5), ABCA3, and CSF2RA/B. GO enrichment analysis of the top 77 genes identified 61 potential PAP-related pathways (Fig. 6). The molecular functions (MF) analysis revealed a concentration of genes associated with protein binding, cytokine activity, cytokine receptor activity, and transcription regulatory region DNA binding. Cellular components (CC), including the extracellular space, receptor complex, and GM-CSF receptor complex, were also highlighted. Biological processes (BP) associated with PAP included signal transduction, the immune response, cytokine-mediated signaling pathways, and cellular responses to lipopolysaccharide. Some important pathways related to lipid metabolism and cholesterol homeostasis were also found.

## Discussion

This global PAP research provides comprehensive information on the clinical phenotypes, prognostic factors related to mortality, and genetic mechanisms associated with PAP. Our findings indicate that the primary clinical manifestations of PAP are respiratory-related, with respiratory failure and pulmonary infections being the primary causes of mortality. There is significant clinical heterogeneity in PAP, with a poorer prognosis in patients with more clinical symptoms. SPAP have a poorer long-term survival rate compared to other types of PAP. Interventions, especially WLL, significantly improved the survival rate. Gene pathway analysis confirmed that the pathogenesis of PAP is complex and suggested that lipid metabolism pathways may be a potential therapeutic target. To our knowledge, this is the first study to systematically assess the impact of the clinical phenotype of PAP on the prognosis and mortality.

PAP is a rare disease characterized by accumulation of surfactant material in the alveoli [16, 17]. Epidemiological studies have mainly focused on prevalence, which is about 6.87 per million in the US and 6.2 per million in Japan [3, 6]. However, research on the prognosis of PAP, including mortality, has been limited to case reports and small clinical cohorts [18–20]. Our present study, which provides clinical information and outcomes for 3278 patients with PAP worldwide, found a 5-year mortality rate of 9.5%. Respiratory complications, particularly respiratory failure and pulmonary infections, were the main causes of death. Meanwhile, the clinical manifestation of PAP is marked by an insidious onset, with its symptoms frequently being non-specific. Its most common symptoms are dyspnea and cough, which are often misdiagnosed [21]. This aligns with our global data and rare disease databases. Furthermore, IPAP and SPAP differ in their clinical focus. IPAP is associated with various comorbidities,leading to missed diagnoses and delayed treatment [22–24]. In contrast, SPAP is considered within the context of secondary factors, mainly hematological disorders [25, 26]. Our analysis confirms that lung infection is the primary comorbidity in IPAP, with bacterial infections, especially by Nocardia, being the most common. This result is consistent with recent research [27]. Additionally, our study provides evidence that hematological disorders are the leading secondary factor in SPAP. These findings highlight the nonspecific and complex nature of clinical features of PAP.

Recent studies have identified a wide range of phenotypes in patients with PAP but their results have been contradictory. Despite the characteristic imaging findings, patients may present with a range of symptoms from severe to mild or even asymptomatic [8–10]. Patients with PAP who present with severe clinical symptoms often show typical radiographic features and reduced oxygen saturation and diffusion capacity [1, 28]. However, there have been reports of patients with severe clinical symptoms presenting with normal pulmonary function indices [8]. Other researchers have also found that patients showing typical radiographic features of PAP may have mild or no symptoms [9, 10]. Asymptomatic patients might have poorer prognoses, while those with severe symptoms can experience stability or spontaneous remission [11–13, 29]. In this study, we again found heterogeneity between phenotypes and outcomes in patients with PAP. Cluster analysis suggests that the mortality rate is higher in patients with a broader spectrum of symptoms of PAP than in their counterparts who are asymptomatic or have only one symptom. Deaths due to respiratory issues, particularly respiratory distress, were more frequent in patients with multiple symptoms, even with increased WLL frequency. Notably, the mortality rate seemed to be higher when the primary symptom was cough rather than isolated dyspnea or respiratory distress alone. The nonspecific and ubiquitous nature of cough may lead to lack of timely attention from both patients and clinicians. Future research should focus on identifying and managing PAP based on clinical phenotype and developing effective interventions. Analyzing different PAP types and their causes of death could help clarify the relationships between clinical outcomes and associated comorbidities or secondary factors.

Most research on the prognosis of PAP has focused on short-term outcomes. Several cohort studies indicate that SPAP has a poorer prognosis compared to IPAP [25, 26], a finding supported by a recent case–control study in Taiwan [30]. Our study also found that SPAP patients have a significantly higher mortality rate than IPAP patients. Additionally, our pooled analysis shows a strong association between SPAP and increased 5-year mortality. Within the SPAP population, patients with SPAP secondary to hematological disorders had higher mortality rates, which due to the underlying hematological disease, disease progression, or associated infections [25, 26]. Typical lesions on high-resolution CT scans, such as a paver-like pattern, were not associated with long-term outcomes. As an additional prognostic factor, therapeutic interventions play a crucial role in PAP outcomes. WLL removes the lipoprotein-like material deposited in the alveolar spaces and is a common first-line treatment for PAP. Studies have shown that WLL can bring about prompt improvement in clinical status and pulmonary function in patients with PAP [14, 31]. However, due to the recurrent nature of PAP symptoms, most patients require repeated WLL treatments [32]. Given the complexity of WLL and its potential complications, GM-CSF therapy has emerged as another option for PAP [33]. Several studies have found that inhaled GM-CSF therapy promotes the improvement and remission of PAP symptoms [34, 35]. However, some reports indicate that neither WLL nor GM-CSF improves long-term prognosis [14, 36, 37]. Our research provides new evidence that WLL and GM-CSF have long-term benefits in these patients: irrespective of type, both initial WLL and repeated WLL significantly reduce the long-term mortality rates. Moreover, GM-CSF therapy significantly lowered the long-term mortality rate in IPAP. Notably, patients who responded well to GM-CSF had lower long-term mortality rates. These findings suggest that the treatment modality may have a significant impact on the long-term prognosis of PAP. Recent studies of GM-CSF therapy in PAP have revealed variations in effectiveness according to disease severity, necessitating identification of potential biomarkers to determine which patients are likely to benefit from GM-CSF [38, 39]. In addition, PAP is characterized by surfactant accumulation within alveoli, with disease severity primarily assessed through diffusing capacity of the lung for carbon monoxide (DLCO) and arterial partial pressure of oxygen (PaO2). Study has demonstrated that PAP patients exhibit significantly decreased DLCO and PaO2 levels, with progressive decline correlating with disease advancement (27,635,117) [40]. A cohort analyses reveal that DLCO exhibits superior prognostic value, showing stronger correlation with disease progression and significant disparity between survival outcomes [20]. These findings indicate that alveolar-capillary membrane diffusion impairment plays a more crucial role in disease progression than hypoxemia alone. Notably, therapeutic whole lung lavage significantly improves both DLCO, PaO2 and clinical outcomes [41, 42] (34,277,049, 20,191,038), underscoring its therapeutic efficacy. Future studies are needed to identify more independent prognostic factors for PAP.

During the past decade, there have been important advances in our understanding of the genetic and mechanistic underpinnings of PAP. Genetic studies have identified common mutations in primary hereditary PAP associated with GM-CSF, particularly at the CSF2RA and CSF2RB loci, and mutations in surfactant-related genes like SFTPC [43]. These findings are consistent with global reports and data from the gene databases. Recent therapeutic strategies for PAP have focused on targeting these genetic and mechanistic pathways [44]. Studies in healthy individuals have shown that surfactants are partly absorbed and metabolized by alveolar macrophages (AMs) mainly via substances such as low-density lipoprotein [45]. Research has revealed that transplanting to restore the activity of AMs might aid in the recovery of surfactant metabolism [46]. Results from two clinical trials indicate that monoclonal antibodies against CD20 can effectively alleviate the clinical symptoms of PAP. [47, 48].

Our pathway enrichment results also provide evidence for cellular response regulation pathways as potential therapeutic targets in PAP. We also discovered that some mechanisms in PAP may relate to metabolism of cholesterol and its homeostasis. This finding could be attributed to disruptions in the GM-CSF signaling pathway leading to impaired function of AMs, affecting the excretion of cholesterol and resulting in accumulation of cholesterol in the AMs in patients with PAP [2]. Our previous research has confirmed the potential role of lipid-related mechanisms in these patients [49]. As a protein convertase, PCSK9 can raise circulating LDL levels by directing liver LDL receptors to lysosomal degradation [50]. PCSK9-mediated LDL elevation was significantly associated with an increased risk of PAP (38,124,156) [49]. One study indicated that oral statins increase excretion of cholesterol and reduce its accumulation in AMs, thereby decreasing the severity of PAP [51]. Therefore, lipid metabolism may play a role in development of PAP. Further research is needed to clarify the direction and efficacy of targeted treatments addressing lipid metabolism in PAP.

This study has several limitations. First, the data were derived from multiple countries and centers, which means that the quality and completeness of the data may be inconsistent. Second, some important covariates may not have been recorded because of the rarity of PAP and the retrospective nature of the data. Nevertheless, our study provides evidence from a large global sample of patients with PAP on the relationship between patient characteristics and mortality, and has identified potential therapeutic directions.

## Conclusions

This study is the first to compile the clinical characteristics, prognosis, and associated phenotypes of PAP based on the largest sample size in the world. Although respiratory failure and lung infections are the most common, the causes of death in PAP patients are diverse and complex, especially those that have not been previously highlighted. PAP exhibits significant clinical heterogeneity, and patients with multiple concurrent clinical conditions demonstrate poorer prognostic outcomes. The impact of the underlying disease on the long-term prognosis of SPAP patients warrants careful consideration. The long-term survival of PAP patients improved significantly after WLL and GM-CSF treatment, and it is necessary to verify the long-term effectiveness of GM-CSF treatment. The targeting mechanism or individualized therapy needs further study.

## Supplementary Information

Supplementary Material 1.Table A1: Literature source information included in the global PAP population study.

Supplementary Material 2.Table A2: Clinical features and phenotypes of autoimmune alveolar proteinosis in the Orphanet and Human Phenotype Ontology database.

Supplementary Material 3.Table A3: Clinical features and phenotypes of hereditary alveolar proteinosis in the Orphanet and Human Phenotype Ontology database.

Supplementary Material 4.Table A4: Clinical characteristics and outcomes of different groups in the PAP cluster of single respiratory symptoms.

Supplementary Material 5.Table A5: Clinical characteristics and outcomes of different groups in the PAP cluster combining two respiratory symptoms.

Supplementary Material 6.Table A6: Cohort information of  211 PAP patients included in pooled analysis.

Supplementary Material 7.Table A7: Characteristics for 211 PAP patients in the pooled analysis.

Supplementary Material 8.Table A8: Information on related gene mutations in the global PAP population study.

Supplementary Material 9.Table A9: Summary of genes and descriptions related to PAP in GeneCards Database.

Supplementary Material 10.Table A10: Summary of genes and descriptions related to Autoimmune PAP in GeneCards Database.

Supplementary Material 11.Table A11: Summary of genes and descriptions related to Secondary PAP in GeneCards Database.

Supplementary Material 12.Table A12: Summary of genes and descriptions related to Hereditary PAP in GeneCards Database.

Supplementary Material 13: Figure S1. Flow chart of studies included in the Global PAP Pooled Analysis.

Supplementary Material 14: Figure S2. Cluster distribution map of Kmeans algorithm based on three respiratory symptoms.

Supplementary Material 15: Figure S3. Histogram of mortality distribution on clustering results. (A)Histogram of mortality distribution for three types based on clustering results; (B) Histogram of mortality distribution in a single symptom group based on clustering results.

Supplementary Material 16: Figure S4. Effect of GM-CSF treatment effectiveness on mortality in PAP population. Abbreviations: GM-CSF, granulocyte-macrophage colony-stimulating factor.

Supplementary Material 17: Figure S5. Heat map of correlation between lavage rate, CT typical lesion rate and cohort mortality.

Supplementary Material 18: Figure S6. Distribution of 211 PAP patients in individual analysis. (A)Distribution of 211 PAP patients by sex and disease type; (B) Age distribution of 211 PAP patients; (C) Age distribution of clinical outcomes grouped by sex; (D) Age distribution of clinical outcomes grouped by disease type. Abbreviations: PAP, pulmonary alveolar proteinosis; SPAP: secondary pulmonary alveolar proteinosis.

Supplementary Material 19: Figure S7. Comparison of different groups in terms of age. (A)Comparison of age distribution of clinical outcomes; (B) Comparison of age distribution of sex; (C) Comparison of age distribution of disease type.

Supplementary Material 20: Figure S8. Comparison of age distribution in survival status between different subgroups. (A) Comparison of age distribution of clinical outcomes in female subgroup; (B) Comparison of age distribution of clinical outcomes in male subgroup; (C) Comparison of age distribution of clinical outcomes in SPAP subgroup; (D) Comparison of age distribution of clinical outcomes in other type of PAP subgroup.

Supplementary Material 21.Detailed methodological information and description.

## Data Availability

To safeguard the privacy of study participants, we cannot openly share the data. However, the datasets utilized or analyzed in this study can be obtained from the corresponding author upon reasonable request. Data are stored in controlled access data storage at the First Hospital of Guangzhou Medical University.

## Abbreviations

PAP: Pulmonary Alveolar Proteinosis
APAP: Autoimmune Pulmonary Alveolar Proteinosis
GM-CSF: Granulocyte-Macrophage Colony-Stimulating Factor
SPAP: Secondary Pulmonary Alveolar Proteinosis
CPAP: Congenital Pulmonary Alveolar Proteinosis
CT: Computed Tomography
WLL: Whole Lung Lavage
IPAP: Idiopathic Pulmonary Alveolar Proteinosis
GO: Gene Ontology
FDR: False Discovery Rate
DLCO: Diffusing Capacity of the Lung for Carbon Monoxide
PaO2: Arterial Partial Pressure of Oxygen
AM: Alveolar Macrophages
LDL: Low-Density Lipoprotein
