# Supplementary material for: Phenotypic heterogeneity in mortality and prognosis of pulmonary alveolar proteinosis: a large-scale, global pooled analysis of individual-level data
Source: Orphanet J Rare Dis. 2025 Mar 4;20:102. doi: 10.1186/s13023-025-03617-3 (PMC11881271; doi:10.1186/s13023-025-03617-3)
Supplement: Supplementary file 1 — Supplementary Material 1.Table A1: Literature source information included in the global PAP population study. [file 13023_2025_3617_MOESM1_ESM.docx]

**Table A1** Literature source information included in the global PAP population study.

| PMID | Author | Year | Country | Patients Number |
| --- | --- | --- | --- | --- |
| 33644180 | Chen | 2021 | China | 1 |
| 33969072 | Wu | 2021 | China | 1 |
| 33678652 | Long | 2021 | China | 25 |
| 32819882 | Yamasaki | 2021 | Japan | 1 |
| 34151899 | Grutters | 2021 | USA | 11 |
| 32528843 | Lawi | 2020 | Switzerland | 1 |
| 32401087 | Papanota | 2020 | Greece | 1 |
| 31718874 | Chandima | 2020 | Australia | 1 |
| 32448830 | Sugiura | 2020 | Japan | 1 |
| 33173585 | Tashiro | 2020 | Japan | 1 |
| 31875636 | Hashimoto | 2020 | Japan | 1 |
| 32358169 | Maite | 2020 | Spain | 1 |
| 32313654 | Suzuki | 2020 | Japan | 1 |
| 32596190 | Zhang, F. | 2020 | Mixed | 1 |
| 31898493 | Zhang, F. | 2020 | China | 20 |
| 32897035 | Trapnell | 2020 | USA | 138 |
| 30113436 | Sirin Kose | 2020 | Turkey | 2 |
| 33257389 | Raziq | 2020 | USA | 1 |
| 33154224 | Punwani | 2020 | India | 1 |
| 32336733 | Nakamura | 2020 | Japan | 1 |
| 32643638 | Marwah | 2020 | India | 10 |
| 31985141 | Kobayashi | 2020 | Japan | 1 |
| 32392077 | Israel | 2020 | USA | 1 |
| 25152273 | Soma | 2019 | India | 1 |
| 29159574 | Sira | 2019 | Spain | 1 |
| 30866900 | Boyce | 2019 | USA | 1 |
| 30295888 | Hirakawa | 2019 | Japan | 1 |
| 31537595 | Inoue | 2019 | Japan | 1 |
| 31712233 | Saha | 2019 | India | 1 |
| 29368649 | Zhang | 2019 | China | 9 |
| 31307910 | Simth | 2019 | USA | 40 |
| 31249778 | Zhang, H. | 2019 | China | 1 |
| 30997074 | Tanaka | 2019 | Japan | 1 |
| 30723866 | Mohammed | 2019 | Kingdom of Saudi Arabia | 1 |
| 29185156 | Tanaka Kubota | 2018 | Japan | 2 |
| 29110133 | Kamboj | 2018 | USA | 1 |
| 30745796 | Moreira | 2018 | Portugal | 1 |
| 29240908 | McElvaney | 2018 | Ireland | 1 |
| 29490416 | Kiani | 2018 | Iranian | 44 |
| 30043890 | Athayde | 2018 | Brazil | 12 |
| 32476928 | Zhang, N. | 2018 | China | 1 |
| 29695229 | Soyez | 2018 | France | 13 |
| 29970780 | Ramachandran | 2018 | India | 1 |
| 29607238 | Li, M. | 2018 | Canada | 1 |
| 26872621 | Imura | 2018 | Japan | 2 |
| 29997992 | Ikeda | 2018 | Japan | 1 |
| 30505485 | Da Nam | 2018 | Korea | 25 |
| 30004070 | Jayaraman | 2018 | India | 2 |
| 27879496 | Altaf | 2017 | USA | 2 |
| 28393663 | Bapat | 2017 | USA | 1 |
| 28270188 | Galhenage | 2017 | Sri Lanka | 1 |
| 28232408 | Philippot | 2017 | France | 4 |
| 28118623 | Gay | 2017 | France | 33 |
| 26148820 | Zhou | 2017 | China | 1 |
| 29095306 | Liu | 2017 | China | 2 |
| 28464852 | Al Haidary | 2017 | Saudi Arabia | 1 |
| 28507378 | Rashid | 2017 | Pakistan | 2 |
| 29162083 | Hwang | 2017 | Korea | 78 |
| 28794843 | Ahn | 2017 | Korea | 1 |
| 28861115 | Jin | 2017 | Korea | 1 |
| 28966213 | Yamasue | 2017 | Japan | 1 |
| 28202867 | Imoto | 2017 | Japan | 1 |
| 28212655 | Masayuki Ito | 2017 | Japan | 1 |
| 28242199 | Ito | 2017 | Japan | 2 |
| 28421817 | Ohkouchi | 2017 | Japan | 5 |
| 28748093 | Ono | 2017 | Japan | 1 |
| 32476827 | Kobayashi | 2017 | Japan | 2 |
| 32476854 | Ayumu | 2017 | Japan | 1 |
| 27783330 | Tanner | 2017 | Finland | 2 |
| 29021953 | Gando | 2017 | Chile | 1 |
| 28718013 | Sira | 2017 | Spain | 1 |
| 28514019 | Uzmezoglu | 2017 | Turkey | 2 |
| 28088396 | Lin, J. | 2017 | USA | 1 |
| 28512724 | Kundović | 2017 | Croatia | 1 |
| 26039225 | Zhang | 2016 | China | 12 |
| 27213073 | Tokman | 2016 | USA | 1 |
| 27548000 | Akira | 2016 | Japan | 44 |
| 27595063 | Takaki | 2016 | Japan | 1 |
| 27052091 | Chauhan | 2016 | India | 1 |
| 27799394 | Ballerie | 2016 | France | 1 |
| 27099254 | Chew | 2016 | Australia | 1 |
| 26843507 | Nagasawa | 2016 | Japan | 1 |
| 28031836 | Huaringa | 2016 | USA | 1 |
| 27366571 | Öneml | 2016 | Turkey | 1 |
| 27833966 | Deleanu | 2016 | Romania | 20 |
| 26519525 | Hiraki | 2016 | Japan | 1 |
| 27890991 | Hadda | 2016 | India | 5 |
| 27408787 | Sideris | 2016 | Greece | 1 |
| 26558331 | Manali | 2016 | Greece | 13 |
| 27445535 | Mo | 2016 | China | 11 |
| 27635117 | Bai | 2016 | China | 101 |
| 27445536 | Kroll | 2016 | Canada | 1 |
| 27512562 | Sha | 2016 | Australia | 1 |
| 25103284 | Antoon | 2016 | USA | 1 |
| 25664978 | Wilson | 2015 | UK | 2 |
| 25940262 | Narotzky | 2015 | USA | 1 |
| 25737738 | Kim | 2015 | Korea | 1 |
| 26317278 | Moriyama | 2015 | Japan | 1 |
| 25925248 | Rodrigo | 2015 | Sri Lanka | 1 |
| 25590481 | Danilevskaya | 2015 | Russia | 6 |
| 26264717 | Akasaka | 2015 | Japan | 31 |
| 26666607 | Arai | 2015 | Japan | 1 |
| 25899759 | Iwakami | 2015 | Japan | 1 |
| 26310609 | Tanaka | 2015 | Japan | 1 |
| 26110014 | Ahmadi | 2015 | Iran | 1 |
| 25814803 | Davis | 2015 | India | 1 |
| 26069841 | Baro | 2015 | india | 1 |
| 26481735 | Zhao | 2015 | China | 120 |
| 25694861 | Zhang | 2015 | China | 1 |
| 26559798 | Fijołek | 2015 | Poland | 1 |
| 25557091 | Garber | 2015 | USA | 1 |
| 26770609 | Guo | 2015 | China | 1 |
| 25335805 | Valimahamed Mitha | 2015 | France | 10 |
| 26621369 | Krecmerova | 2015 | Czech Republic | 1 |
| 29043131 | Sewaralthahab | 2015 | USA | 1 |
| 25534398 | Bonella | 2015 | Mixed | 31 |
| 26264606 | Griese | 2015 | Germany | 21 |
| 25580277 | Lopez | 2014 | Switzerland | 1 |
| 24793148 | Fijołek | 2014 | Poland | 17 |
| 25300566 | Chaulagain | 2014 | USA | 1 |
| 24977032 | Chan | 2014 | Singapore | 1 |
| 25042291 | Silva | 2014 | Portugal | 3 |
| 24388375 | Handa | 2014 | Japan | 3 |
| 23749726 | McDonnell | 2014 | Ireland | 2 |
| 24927752 | Enaud | 2014 | France | 34 |
| 25117372 | Gao | 2014 | China | 1 |
| 24886114 | Yu | 2014 | China | 1 |
| 25120596 | Zhou | 2014 | China | 11 |
| 24859540 | Strickler | 2014 | Chile | 1 |
| 25361924 | Ilkovich | 2014 | Russia | 68 |
| 25139636 | Sawai | 2014 | Japan | 1 |
| 25125821 | Hasan | 2014 | India | 1 |
| 23645284 | Raj | 2014 | India | 1 |
| 25366193 | Kinehara | 2014 | Japan | 1 |
| 24597668 | Ishii | 2014 | Japan | 31 |
| 24726085 | Ferreira | 2014 | Portugal | 1 |
| 24707326 | Mehrian P | 2014 | Iran | 35 |
| 23905534 | Marchand Adam | 2013 | France | 1 |
| 24339646 | Bansal | 2013 | USA | 1 |
| 23632425 | Stafford | 2013 | USA | 1 |
| 23710403 | Hammami | 2013 | Tunisia | 1 |
| 23550265 | Badiozaman | 2013 | Iran | 9 |
| 24532945 | Bhattacharyya | 2013 | India | 2 |
| 23523160 | Moisan | 2013 | France | 1 |
| 23497546 | Campo | 2013 | Italy | 73 |
| 24162118 | Baldi | 2013 | India | 1 |
| 23886640 | Nagasawa | 2013 | Japan | 1 |
| 23328142 | Hasan | 2013 | USA | 1 |
| 25473545 | Zhao | 2013 | China | 1 |
| 26029499 | Main | 2013 | UK | 1 |
| 23821516 | Albores | 2013 | USA | 1 |
| 21621361 | Guan | 2012 | China | 38 |
| 26057858 | Tekgül | 2012 | Turkey | 1 |
| 22553271 | Compa | 2012 | USA | 1 |
| 22495262 | Kumar | 2012 | USA | 2 |
| 23049634 | Stoica | 2012 | Romania | 1 |
| 21800116 | Park | 2012 | Korea | 1 |
| 22627079 | Vanderhelst | 2012 | Germany | 1 |
| 22543305 | Cai | 2012 | China | 1 |
| 21395954 | Ansari | 2012 | Canada | 2 |
| 22440382 | Gonçalves | 2012 | Brazil | 1 |
| 23176995 | Rebelo | 2012 | Brazil | 1 |
| 22484272 | Punatar | 2012 | USA | 1 |
| 22973777 | Khan | 2012 | India | 5 |
| 22365249 | Garcia | 2011 | USA | 1 |
| 21884300 | Nishino | 2011 | Japan | 5 |
| 21478218 | Kavuru | 2011 | USA | 9 |
| 21873931 | Lingadevaru | 2011 | USA | 1 |
| 21217786 | Pidala | 2011 | USA | 1 |
| 22977542 | Firat | 2011 | Turkey | 2 |
| 21655022 | El Dawlatly | 2011 | KSA | 1 |
| 21513605 | Luisetti | 2011 | Italy | 2 |
| 24765314 | Nicolini | 2011 | Italy | 1 |
| 21900000 | Bonella | 2011 | Germany | 70 |
| 21849033 | Griese | 2011 | Germany | 1 |
| 21284854 | Tejwani | 2011 | USA | 1 |
| 21773908 | Tagawa | 2011 | Japan | 1 |
| 21600085 | Huang | 2011 | China | 9 |
| 21282812 | H Ishii | 2011 | Japan | 404 |
| 20117952 | Lin | 2010 | China | 1 |
| 20167854 | Tazawa | 2010 | Japan | 50 |
| 22958601 | Tetikkurt | 2010 | Turkey | 1 |
| 20623200 | Shah | 2010 | USA | 1 |
| 21037373 | Edwards | 2010 | UK | 1 |
| 19955712 | Xue | 2010 | China | 1 |
| 20622029 | Suzuki | 2010 | USA | 8 |
| 20453607 | DiBlasi | 2010 | USA | 1 |
| 21038791 | Jirarattanasopa | 2010 | Thailand | 1 |
| 20191038 | Byun | 2010 | Korea | 38 |
| 20034968 | Hodges | 2010 | South Africa | 1 |
| 20855439 | Amital | 2010 | Israel | 1 |
| 21526069 | Tabatabaei | 2010 | Iran | 8 |
| 20539770 | Jayaraman | 2010 | India | 1 |
| 21199121 | Reiter | 2010 | Germany | 4 |
| 20019344 | Cummings | 2010 | USA | 2 |
| 20484303 | Kadikoy | 2010 | USA | 1 |
| 20185750 | Costabel | 2010 | Mixed | 2 |
| 19483052 | Borie | 2009 | France | 1 |
| 19210651 | Uchiyama | 2009 | Japan | 1 |
| 19693450 | Tabata | 2009 | Japan | 1 |
| 21886658 | McDermott | 2009 | UK | 1 |
| 19329018 | Doğru | 2009 | Turkey | 1 |
| 19407056 | Luisetti | 2009 | Italy | 1 |
| 20532003 | Thind | 2009 | India | 1 |
| 20640150 | Nandkumar | 2009 | India | 1 |
| 19781325 | Zhou | 2009 | China | 5 |
| 21686581 | Figueiredo | 2009 | Beazil | 1 |
| 19265094 | Robinson | 2009 | Germany | 2 |
| 19465834 | Chung | 2009 | USA | 6 |
| 19892674 | Haruyuki | 2009 | Japan | 42 |
| 21318986 | Ohmachi | 2008 | Japan | 1 |
| 19038019 | Webb | 2008 | UK | 1 |
| 18618617 | Yamamoto | 2008 | Japan | 1 |
| 17337244 | Sihoe | 2008 | China | 1 |
| 18085671 | Inaba | 2008 | USA | 5 |
| 18496859 | Patiroglu | 2008 | Turkey | 1 |
| 18955567 | MartinezunknowMoczygemba | 2008 | USA | 1 |
| 18551202 | Juvet | 2008 | Canada | 1 |
| 18202348 | Inoue | 2008 | Japan | 248 |
| 17386098 | Ceruti | 2007 | Italy | 1 |
| 17242125 | Kumar | 2007 | India | 1 |
| 17244596 | Sauni | 2007 | Finland | 1 |
| 17291222 | Pedroso | 2007 | France | 1 |
| 17827846 | Yamada | 2007 | Japan | 1 |
| 17256566 | Indira | 2007 | India | 1 |
| 16806874 | Froudarakis | 2007 | Greece | 1 |
| 17566600 | Su | 2007 | China | 2 |
| 16953205 | Fukuno | 2006 | Japan | 1 |
| 16840407 | Venkateshiah | 2006 | USA | 25 |
| 16475176 | Price | 2006 | Canada | 1 |
| 16906593 | Pollack | 2006 | USA | 1 |
| 16432866 | Numata | 2006 | Japan | 1 |
| 16517574 | Lin | 2006 | China | 16 |
| 16507860 | Wylam | 2006 | USA | 12 |
| 16916345 | Noel R Wardwell Jr | 2006 | USA | 1 |
| 17273616 | Thompson | 2006 | Portugal | 4 |
| 16423273 | Yoshikazu Inoue | 2006 | Japan | 166 |
| 15912060 | KoplinunknowBaucum | 2005 | USA | 1 |
| 15735059 | Tazawa | 2005 | Japan | 3 |
| 15756373 | Ahmed | 2005 | Saudi Arabia | 1 |
| 15569500 | Cai | 2005 | China | 1 |
| 17670454 | Centella | 2005 | Spain | 1 |
| 15333392 | Nadeau | 2004 | Canada | 1 |
| 15497254 | Rahman | 2004 | Australia | 1 |
| 15648606 | Akin | 2004 | USA | 3 |
| 15588045 | Arai | 2004 | Japan | 1 |
| 15083749 | Beccaria | 2004 | Italy | 21 |
| 14734139 | Gal | 2004 | USA | 1 |
| 15519209 | Kim | 2004 | Korea | 1 |
| 15189962 | Perez | 2004 | USA | 5 |
| 15289783 | Santamaria | 2004 | Italy | 1 |
| 15730782 | Xu KF | 2004 | China | 17 |
| 14512323 | Uchida K | 2004 | Japan | 107 |
| 12802928 | Pamuk | 2003 | Turkey | 1 |
| 12636240 | Ohnishi | 2003 | Japan | 1 |
| 14587059 | Goldschmidt | 2003 | Israel | 1 |
| 14656624 | Wagner | 2003 | Germany | 1 |
| 12715332 | Arbiser | 2003 | USA | 2 |
| 14753379 | Horton | 2003 | USA | 2 |
| 12854908 | Tsutsumi | 2003 | Japan | 1 |
| 12663343 | Kavuru | 2003 | USA | 1 |
| 14614982 | Calderazzo | 2003 | Italy | 1 |
| 12832691 | Khanjari | 2003 | Mixed | 1 |
| 12612307 | Seymour | 2003 | Australia | 12 |
| 14567558 | Thomassen | 2003 | USA | 5 |
| 12508267 | Vella | 2003 | Italy | 1 |
| 12498816 | Bonfield | 2002 | USA | 14 |
| 12200534 | de Vega | 2002 | Spain | 1 |
| 12119235 | Seymour | 2002 | Mixed | 343 |
| 12521210 | Hashizume | 2002 | Japan | 1 |
| 12058888 | Ishikawa | 2002 | Japan | 1 |
| 12007507 | Shoji | 2002 | Japan | 4 |
| 12111790 | Tomonari | 2002 | Japan | 1 |
| 11805749 | BenunknowAbraham | 2002 | Israel | 5 |
| 12377884 | Cheng | 2002 | China | 3 |
| 12547151 | Ando | 2002 | Japan | 1 |
| 12356582 | Bonfield | 2002 | USA | 40 |
| 12475870 | Cheng | 2002 | China | 1 |
| 12153694 | Yoshioka | 2002 | Japan | 1 |
| 11685098 | Du | 2001 | USA | 1 |
| 11312196 | Holbert | 2001 | USA | 27 |
| 11688827 | Minakata | 2001 | Japan | 1 |
| 11171752 | Mildenberger | 2001 | Germany | 2 |
| 11179134 | Seymour | 2001 | Mixed | 14 |
| 10764303 | Kavuru | 2000 | USA | 4 |
| 10781712 | Birsak | 2000 | Netherlands | 1 |
| 10331561 | Kim | 1999 | Korea | 12 |
| 10464867 | Kadota | 1999 | Japan | 1 |
| 9565812 | Gacouin | 1998 | France | 1 |
| 8810625 | Alberti | 1996 | Italy | 5 |
| 1774397 | Aguinaga | 1991 | Spain | 2 |
| 6792945 | Harrison | 1981 | Australia | 2 |
| 921378 | Selecky | 1977 | USA | 19 |
| 5027640 | Ganguli | 1972 | Canada | 1 |
| 9476887 | Davidson | 1969 | USA | 21 |
