# Supplementary material for: Phenotypic heterogeneity in mortality and prognosis of pulmonary alveolar proteinosis: a large-scale, global pooled analysis of individual-level data
Source: Orphanet J Rare Dis. 2025 Mar 4;20:102. doi: 10.1186/s13023-025-03617-3 (PMC11881271; doi:10.1186/s13023-025-03617-3)
Supplement: Supplementary file 2 — Supplementary Material 2.Table A2: Clinical features and phenotypes of autoimmune alveolar proteinosis in the Orphanet and Human Phenotype Ontology database. [file 13023_2025_3617_MOESM2_ESM.docx]

**Table A2** Clinical features and phenotypes of autoimmune alveolar proteinosis in the Orphanet and Human Phenotype Ontology database.

| HPO_TERM_ID | HPO_TERM_NAME | CATEGORY | HPO Frequency |
| --- | --- | --- | --- |
| HP:0006517 | Intraalveolar phospholipid accumulation | Respiratory System | Very frequent (80-99%) |
| HP:0002087 | Abnormality of the upper respiratory tract | Respiratory System | Frequent (30-79%) |
| HP:0002091 | Restrictive ventilatory defect | Respiratory System | Frequent (30-79%) |
| HP:0002094 | Dyspnea | Respiratory System | Frequent (30-79%) |
| HP:0012418 | Hypoxemia | Respiratory System | Frequent (30-79%) |
| HP:0045051 | Decreased DLCO | Respiratory System | Frequent (30-79%) |
| HP:0012735 | Cough | Respiratory System | Occasional (5-29%) |
| HP:0025391 | Crazy paving pattern | Respiratory System | Occasional (5-29%) |
| HP:0030830 | Crackles | Respiratory System | Occasional (5-29%) |
| HP:0002105 | Hemoptysis | Respiratory System | Very rare (1-<4%) |
| HP:0030057 | Autoimmune antibody positivity | Immunology | Frequent (30-79%) |
| HP:0000961 | Cyanosis | Skin, Hair, and Nails | Frequent (30-79%) |
| HP:0001217 | Clubbing | Skeletal system | Frequent (30-79%) |
| HP:0025435 | Increased circulating lactate dehydrogenase concentration | Metabolism/Laboratory abnormality | Frequent (30-79%) |
| HP:0010876 | Abnormal circulating protein concentration | Metabolism/Laboratory abnormality | Frequent (30-79%) |
| HP:0001945 | Fever | Metabolism/Laboratory abnormality | Very rare (1-<4%) |
| HP:0012378 | Fatigue | Constitutional Symptom | Very rare (1-<4%) |
| HP:0100749 | Chest pain | Constitutional Symptom | Very rare (1-<4%) |
| HP:0003651 | Foam cells | Cardiovascular | Frequent (30-79%) |
| HP:0001824 | Weight loss | Growth | Very rare (1-<4%) |

Abbreviations: HPO, human phenotype ontology; DLCO, lung diffusing capacity for carbon monoxide.
