# Supplementary material for: Phenotypic heterogeneity in mortality and prognosis of pulmonary alveolar proteinosis: a large-scale, global pooled analysis of individual-level data
Source: Orphanet J Rare Dis. 2025 Mar 4;20:102. doi: 10.1186/s13023-025-03617-3 (PMC11881271; doi:10.1186/s13023-025-03617-3)
Supplement: Supplementary file 3 — Supplementary Material 3.Table A3: Clinical features and phenotypes of hereditary alveolar proteinosis in the Orphanet and Human Phenotype Ontology database. [file 13023_2025_3617_MOESM3_ESM.docx]

**Table A3** Clinical features and phenotypes of hereditary alveolar proteinosis in the Orphanet and Human Phenotype Ontology database.

| HPO_TERM_ID | HPO_TERM_NAME | CATEGORY | HPO Frequency |
| --- | --- | --- | --- |
| HP:0002091 | Restrictive ventilatory defect | Respiratory System | Frequent (30-79%) |
| HP:0002098 | Respiratory distress | Respiratory System | Frequent (30-79%) |
| HP:0004887 | Respiratory failure requiring assisted ventilation | Respiratory System | Frequent (30-79%) |
| HP:0012418 | Hypoxemia | Respiratory System | Frequent (30-79%) |
| HP:0025391 | Crazy paving pattern | Respiratory System | Frequent (30-79%) |
| HP:0002789 | Tachypnea | Respiratory System | Occasional (5-29%) |
| HP:0011949 | Acute infectious pneumonia | Respiratory System | Occasional (5-29%) |
| HP:0012735 | Cough | Respiratory System | Occasional (5-29%) |
| HP:0030830 | Crackles | Respiratory System | Occasional (5-29%) |
| HP:0010876 | Abnormal circulating protein concentration | Metabolism/Laboratory abnormality | Very frequent (80-99%) |
| HP:0031029 | Elevated carcinoembryonic antigen level | Metabolism/Laboratory abnormality | Occasional (5-29%) |
| HP:0001531 | Failure to thrive in infancy | Growth | Frequent (30-79%) |
| HP:0001649 | Tachycardia | Cardiovascular | Occasional (5-29%) |
| HP:0003651 | Foam cells | Cardiovascular | Occasional (5-29%) |
| HP:0030057 | Autoimmune antibody positivity | Immunology | Occasional (5-29%) |

Abbreviations: HPO, human phenotype ontology.
