# Supplementary material for: Phenotypic heterogeneity in mortality and prognosis of pulmonary alveolar proteinosis: a large-scale, global pooled analysis of individual-level data
Source: Orphanet J Rare Dis. 2025 Mar 4;20:102. doi: 10.1186/s13023-025-03617-3 (PMC11881271; doi:10.1186/s13023-025-03617-3)
Supplement: Supplementary file 4 — Supplementary Material 4.Table A4: Clinical characteristics and outcomes of different groups in the PAP cluster of single respiratory symptoms. [file 13023_2025_3617_MOESM4_ESM.docx]

**Table A4** Clinical characteristics and outcomes of different groups in the PAP cluster of single respiratory symptoms.

|  | Group | | | P value | | |
| --- | --- | --- | --- | --- | --- | --- |
| Characteristic | C2(N=88) | C3(N=22) | C5(N=436) | C2 vs C3 | C2 vs C5 | C3 vs C5 |
| Number of Death(%) | 17(19.32) | 2(9.09) | 28(6.42) | 0.354 | <0.001 | 0.648 |
| Number of deaths attributed to the Respiratory System(%) | 12(70.59) | 2(100) | 20(71.43) | >0.999 | >0.999 | >0.999 |
| Number of deaths attributed to Respiratory failure(%) | 9(75.00) | 1(50.00) | 12(60.00) | >0.999 | 0.727 | >0.999 |
| Number of deaths attributed to Lung Infections(%) | 3(25.00) | 1(50.00) | 8(40.00) | 0.386 | 0.493 | 0.517 |
| Sex Male(%) | 53(60.23) | 14(63.64) | 259(59.40) | 0.961 | 0.712 | >0.999 |
| PAP Type: Primary(%) | 69(78.41) | 13(59.09) | 363(83.26) | 0.113 | 0.349 | <0.001 |
| Frequency of alveolar lavage | 0.31(0.44) | 0.81(0.33) | 0.65(0.46) | 0.005 | 0.020 | 0.331 |
| Frequency of repeated lung lavage | 0.07(0.26) | 0.62(0.46) | 0.43(0.48) | 0.001 | 0.004 | 0.185 |

1. C2: single cough group; C3, single shortness of breath group; C5, Single dyspnea group.
2. T-test or wilcoxon signed-rank test was used for continuous variables; Chi-square test was used for categorical variables.
