# Supplementary material for: Phenotypic heterogeneity in mortality and prognosis of pulmonary alveolar proteinosis: a large-scale, global pooled analysis of individual-level data
Source: Orphanet J Rare Dis. 2025 Mar 4;20:102. doi: 10.1186/s13023-025-03617-3 (PMC11881271; doi:10.1186/s13023-025-03617-3)
Supplement: Supplementary file 5 — Supplementary Material 5.Table A5: Clinical characteristics and outcomes of different groups in the PAP cluster combining two respiratory symptoms. [file 13023_2025_3617_MOESM5_ESM.docx]

**Table A5** Clinical characteristics and outcomes of different groups in the PAP cluster combining two respiratory symptoms.

|  | Group | | P value |
| --- | --- | --- | --- |
| Characteristic | C1(N=26) | C6(N=1017) | C1vsC6 |
| Number of Death(%) | 13(50.00) | 131(12.88) | <0.001 |
| Number of deaths attributed to the Respiratory System(%) | 6(46.16) | 99(75.57) | 0.044 |
| Number of deaths attributed to Respiratory failure(%) | 6(100) | 77(77.78) | 0.559 |
| Number of deaths attributed to Lung Infections(%) | 0 | 22(22.22) | 0.219 |
| Sex Male(%) | 13(50.00) | 713(70.11) | 0.062 |
| PAP Type: Primary(%) | 4(15.38) | 931(91.54) | <0.001 |
| Frequency of alveolar lavage | 0.54(0.50) | 0.66(0.45) | 0.435 |
| Frequency of repeated lung lavage | 0.28(0.41) | 0.36(0.46) | 0.584 |

1. C1: cough and shortness of breath group; C6, cough and dyspnea group.
2. T-test or wilcoxon signed-rank test was used for continuous variables; Chi-square test was used for categorical variables.
