# Supplementary material for: Phenotypic heterogeneity in mortality and prognosis of pulmonary alveolar proteinosis: a large-scale, global pooled analysis of individual-level data
Source: Orphanet J Rare Dis. 2025 Mar 4;20:102. doi: 10.1186/s13023-025-03617-3 (PMC11881271; doi:10.1186/s13023-025-03617-3)
Supplement: Supplementary file 6 — Supplementary Material 6.Table A6: Cohort information of 211 PAP patients included in pooled analysis. [file 13023_2025_3617_MOESM6_ESM.docx]

**Table A6** Cohort information of 211 PAP patients included in pooled analysis.

| PMID | Patients | Age | Sex |
| --- | --- | --- | --- |
| 32819882 | 1 | 70 | Female |
| 32528843 | 2 | 30 | Female |
| 32401087 | 3 | 79 | Male |
| 31718874 | 4 | 33 | Male |
| 32448830 | 5 | 70 | Male |
| 33173585 | 6 | 29 | Female |
| 32596190 | 7 | 0.75 | Male |
| 33257389 | 8 | 29 | Female |
| 33154224 | 9 | 35 | Male |
| 32336733 | 10 | 78 | Male |
| 31985141 | 11 | 14 | Female |
| 32392077 | 12 | 20 | Female |
| 25152273 | 13 | 37 | Female |
| 30866900 | 14 | 36 | Female |
| 31249778 | 15 | 45 | Male |
| 30997074 | 16 | 58 | Female |
| 30723866 | 17 | 0.5 | Male |
| 29110133 | 18 | 52 | Male |
| 30745796 | 19 | 44 | Male |
| 29240908 | 20 | 43 | Male |
| 32476928 | 21 | 47 | Male |
| 29970780 | 22 | 55 | Female |
| 29607238 | 23 | 47 | Male |
| 29997992 | 24 | 40 | Male |
| 28393663 | 25 | 52 | Female |
| 28270188 | 26 | 15 | Female |
| 26148820 | 27 | 42 | Male |
| 28464852 | 28 | 5 | Male |
| 28794843 | 29 | 30 | Male |
| 28861115 | 30 | 11 | Male |
| 28966213 | 31 | 64 | Female |
| 28202867 | 32 | 69 | Male |
| 28212655 | 33 | 77 | Female |
| 28748093 | 34 | 46 | Female |
| 32476854 | 35 | 46 | Male |
| 29021953 | 36 | 24 | Male |
| 28088396 | 37 | 1.75 | Female |
| 28512724 | 38 | 10 | Male |
| 27213073 | 39 | 67 | Male |
| 27595063 | 40 | 36 | Female |
| 27052091 | 41 | 45 | Female |
| 27799394 | 42 | 28 | Female |
| 27099254 | 43 | 46 | Male |
| 26843507 | 44 | 26 | Female |
| 28031836 | 45 | 52 | Male |
| 27366571 | 46 | 37 | Male |
| 26519525 | 47 | 50 | Male |
| 27408787 | 48 | 8 | Female |
| 27445536 | 49 | 55 | Female |
| 27512562 | 50 | 29 | Female |
| 25103284 | 51 | 16 | Female |
| 25940262 | 52 | 25 | Female |
| 25737738 | 53 | 52 | Male |
| 26317278 | 54 | 44 | Female |
| 25925248 | 55 | 61 | Female |
| 26666607 | 56 | 59 | Male |
| 25899759 | 57 | 78 | Female |
| 26310609 | 58 | 39 | Female |
| 26110014 | 59 | 5 | Male |
| 25814803 | 60 | 33 | Female |
| 26069841 | 61 | 10 | Female |
| 25694861 | 62 | 47 | Male |
| 26559798 | 63 | 31 | Female |
| 25557091 | 64 | 40 | Male |
| 26770609 | 65 | 39 | Male |
| 26621369 | 66 | 45 | Female |
| 29043131 | 67 | 42 | Female |
| 25300566 | 68 | 69 | Male |
| 24977032 | 69 | 68 | Female |
| 25117372 | 70 | 40 | Male |
| 24886114 | 71 | 47 | Female |
| 24859540 | 72 | 13 | Female |
| 25139636 | 73 | 48 | Female |
| 25125821 | 74 | 36 | Female |
| 23645284 | 75 | 8 | Male |
| 25366193 | 76 | 75 | Male |
| 24726085 | 77 | 61 | Male |
| 24339646 | 78 | 54 | Male |
| 23632425 | 79 | 32 | Male |
| 23710403 | 80 | 3 | Male |
| 23523160 | 81 | 27 | Female |
| 24162118 | 82 | 38 | Male |
| 23886640 | 83 | 26 | Female |
| 23328142 | 84 | 44 | Female |
| 25473545 | 85 | 34 | Male |
| 26029499 | 86 | 79 | Female |
| 23821516 | 87 | 46 | Female |
| 26057858 | 88 | 46 | Male |
| 22553271 | 89 | 32 | Female |
| 23049634 | 90 | 34 | Male |
| 21800116 | 91 | 45 | Female |
| 22627079 | 92 | 51 | Male |
| 22543305 | 93 | 39 | Male |
| 22440382 | 94 | 6 | Female |
| 23176995 | 95 | 50 | Female |
| 22484272 | 96 | 65 | Male |
| 21873931 | 97 | 12 | Female |
| 21217786 | 98 | 40 | Female |
| 21655022 | 99 | 15 | Female |
| 24765314 | 100 | 41 | Female |
| 21849033 | 101 | 2.5 | Female |
| 21284854 | 102 | 42 | Male |
| 21773908 | 103 | 43 | Female |
| 20623200 | 104 | 67 | Female |
| 21037373 | 105 | 3 | Male |
| 19955712 | 106 | 41 | Female |
| 20453607 | 107 | 13 | Male |
| 21038791 | 108 | 0.75 | Female |
| 20034968 | 109 | 0.3 | Male |
| 20855439 | 110 | 40 | Female |
| 20539770 | 111 | 26 | Male |
| 20484303 | 112 | 49 | Female |
| 19210651 | 113 | 39 | Female |
| 19693450 | 114 | 48 | Male |
| 21886658 | 115 | 42 | Male |
| 19329018 | 116 | 4 | Male |
| 19407056 | 117 | 40 | Male |
| 20640150 | 118 | 43 | Male |
| 21686581 | 119 | 54 | Male |
| 19038019 | 120 | 34 | Male |
| 18618617 | 121 | 9 | Female |
| 17337244 | 122 | 29 | Female |
| 18496859 | 123 | 16 | Female |
| 18955567 | 124 | 3 | Female |
| 18551202 | 125 | 46 | Female |
| 17256566 | 126 | 53 | Male |
| 16806874 | 127 | 13 | Female |
| 16475176 | 128 | 13 | Female |
| 16432866 | 129 | 43 | Male |
| 17670454 | 130 | 33 | Male |
| 15333392 | 131 | 30 | Male |
| 15497254 | 132 | 44 | Male |
| 15588045 | 133 | 57 | Female |
| 14734139 | 134 | 43 | Male |
| 15519209 | 135 | 59 | Male |
| 15289783 | 136 | 1.7 | Male |
| 12802928 | 137 | 47 | Female |
| 12636240 | 138 | 47 | Male |
| 14587059 | 139 | 64 | Female |
| 14656624 | 140 | 29 | Female |
| 12854908 | 141 | 55 | Female |
| 12663343 | 142 | 41 | Female |
| 14614982 | 143 | 59 | Male |
| 12832691 | 144 | 35 | Male |
| 12508267 | 145 | 64 | Male |
| 12200534 | 146 | 47 | Female |
| 12521210 | 147 | 79 | Female |
| 12058888 | 148 | 48 | Male |
| 12111790 | 149 | 45 | Female |
| 12547151 | 150 | 50 | Male |
| 12475870 | 151 | 29 | Male |
| 12153694 | 152 | 66 | Male |
| 11685098 | 153 | 64 | Male |
| 11688827 | 154 | 48 | Male |
| 10781712 | 155 | 57 | Female |
| 10464867 | 156 | 67 | Male |
| 9565812 | 157 | 26 | Male |
| 5027640 | 158 | 21 | Male |
| 30113436 | 159 | 13 | Male |
|  | 160 | 15 | Female |
| 29185156 | 161 | 0.08 | Female |
|  | 162 | 0.5 | Female |
| 26872621 | 163 | 58 | Female |
|  | 164 | 58 | Female |
| 30004070 | 165 | 0.67 | Male |
|  | 166 | 4 | Male |
|  | 167 | 63 | Male |
|  | 168 | 65 | Male |
| 29095306 | 169 | 39 | Male |
|  | 170 | 65 | Female |
|  | 171 | 24 | Male |
| 32476827 | 172 | 40 | Female |
| 27783330 | 173 | 1 | Female |
| 27890991 | 174 | 27 | Female |
|  | 175 | 32 | Male |
|  | 176 | 30 | Female |
|  | 177 | 47 | Female |
|  | 178 | 71 | Female |
| 25664978 | 179 | 8 | Male |
|  | 180 | 4 | Female |
| 25042291 | 181 | 39 | Male |
|  | 182 | 47 | Female |
|  | 183 | 45 | Male |
|  | 184 | 50 | Female |
| 23749726 | 185 | 52 | Male |
| 24532945 | 186 | 33 | Male |
|  | 187 | 28 | Male |
| 21884300 | 188 | 55 | Male |
|  | 189 | 53 | Female |
|  | 190 | 24 | Male |
|  | 191 | 18 | Female |
|  | 192 | 57 | Male |
| 22977542 | 193 | 31 | Female |
|  | 194 | 44 | Female |
| 20019344 | 195 | 49 | Male |
|  | 196 | 39 | Male |
|  | 197 | 19 | Female |
|  | 198 | 59 | Female |
| 17273616 | 199 | 26 | Female |
|  | 200 | 34 | Female |
|  | 201 | 27 | Female |
| 12715332 | 202 | 36 | Male |
| 12377884 | 203 | 36 | Male |
|  | 204 | 42 | Female |
|  | 205 | 54 | Male |
| 10764303 | 206 | 43 | Male |
|  | 207 | 40 | Male |
|  | 208 | 18 | Male |
|  | 209 | 36 | Male |
| 6792945 | 210 | 52 | Male |
|  | 211 | 44 | Male |
