# Supplementary material for: Phenotypic heterogeneity in mortality and prognosis of pulmonary alveolar proteinosis: a large-scale, global pooled analysis of individual-level data
Source: Orphanet J Rare Dis. 2025 Mar 4;20:102. doi: 10.1186/s13023-025-03617-3 (PMC11881271; doi:10.1186/s13023-025-03617-3)
Supplement: Supplementary file 7 — Supplementary Material 7.Table A7: Characteristics for 211 PAP patients in the pooled analysis. [file 13023_2025_3617_MOESM7_ESM.docx]

**Table A7** Characteristics for 211 PAP patients in the pooled analysis.

|  | Total(N=211) | Alive(N=170) | Death(N=41) | P |
| --- | --- | --- | --- | --- |
| Age group |  |  |  | 0.03 |
| <50 | 154 | 130 | 24 |  |
| >=50 | 57 | 40 | 17 |  |
| Sex group |  |  |  | 0.94 |
| Female | 102 | 83 | 19 |  |
| Male | 109 | 87 | 22 |  |
| Alveolar lavage therapy |  |  |  | <0.001 |
| Yes | 145 | 131 | 14 |  |
| No | 66 | 39 | 27 |  |
| Repeated alveolar lavage therapy |  |  |  | 0.003 |
| Yes | 102 | 91 | 11 |  |
| No | 109 | 79 | 30 |  |
| PAP type |  |  |  | 0.004 |
| SPAP | 90 | 64 | 26 |  |
| Other | 121 | 106 | 15 |  |

1. Chi-square test was used for categorical variables.
2. Abbreviations: SPAP, secondary pulmonary alveolar proteinosis.
