# Supplementary material for: Phenotypic heterogeneity in mortality and prognosis of pulmonary alveolar proteinosis: a large-scale, global pooled analysis of individual-level data
Source: Orphanet J Rare Dis. 2025 Mar 4;20:102. doi: 10.1186/s13023-025-03617-3 (PMC11881271; doi:10.1186/s13023-025-03617-3)
Supplement: Supplementary file 8 — Supplementary Material 8.Table A8: Information on related gene mutations in the global PAP population study. [file 13023_2025_3617_MOESM8_ESM.docx]

**Table A8** Information on related gene mutations in the global PAP population study.

| Year | Number of Patients(n) | Mutant gene |
| --- | --- | --- |
| 2020 | 1 | GATA-2 |
| 2019 | 1 | MARS |
| 2018 | 1 | MASP2 |
| 2017 | 1 | CSF2RA |
| 2017 | 1 | ABCA3 |
| 2017 | 1 | CSF2RA |
| 2016 | 1 | SMPD1 |
| 2015 | 4 | GATA-2 |
| 2014 | 2 | SFTPC |
| 2011 | 1 | CSF2RA |
| 2010 | 1 | CSF2RA |
| 2008 | 1 | X-PAR1 |
| 2001 | 2 | SP-B messenger RNA |
