# Supplementary material for: Phenotypic heterogeneity in mortality and prognosis of pulmonary alveolar proteinosis: a large-scale, global pooled analysis of individual-level data
Source: Orphanet J Rare Dis. 2025 Mar 4;20:102. doi: 10.1186/s13023-025-03617-3 (PMC11881271; doi:10.1186/s13023-025-03617-3)
Supplement: Supplementary file 9 — Supplementary Material 9.Table A9: Summary of genes and descriptions related to PAP in GeneCards Database. [file 13023_2025_3617_MOESM9_ESM.docx]

**Table A9** Summary of genes and descriptions related to PAP in GeneCards Database.

| Gene Symbol | Description | Category | Relevance score |
| --- | --- | --- | --- |
| MARS1 | Methionyl-TRNA Synthetase 1 | Protein Coding | 152.35 |
| ABCA3 | ATP Binding Cassette Subfamily A Member 3 | Protein Coding | 116.96 |
| SFTPB | Surfactant Protein B | Protein Coding | 99.21 |
| SFTPC | Surfactant Protein C | Protein Coding | 91.70 |
| OAS1 | 2'-5'-Oligoadenylate Synthetase 1 | Protein Coding | 74.54 |
| CSF2RA | Colony Stimulating Factor 2 Receptor Subunit Alpha | Protein Coding | 58.62 |
| CSF2RB | Colony Stimulating Factor 2 Receptor Subunit Beta | Protein Coding | 54.38 |
| HLA-DRB1 | Major Histocompatibility Complex, Class II, DR Beta 1 | Protein Coding | 43.78 |
| GATA2 | GATA Binding Protein 2 | Protein Coding | 30.38 |
| FARSB | Phenylalanyl-TRNA Synthetase Subunit Beta | Protein Coding | 29.78 |
| ARHGAP9 | Rho GTPase Activating Protein 9 | Protein Coding | 21.72 |
| CSF2 | Colony Stimulating Factor 2 | Protein Coding | 21.37 |
| SFTPD | Surfactant Protein D | Protein Coding | 18.55 |
| SLC7A7 | Solute Carrier Family 7 Member 7 | Protein Coding | 13.53 |
| SPI1 | Spi-1 Proto-Oncogene | Protein Coding | 12.83 |
| CCL2 | C-C Motif Chemokine Ligand 2 | Protein Coding | 12.33 |
| ABCG1 | ATP Binding Cassette Subfamily G Member 1 | Protein Coding | 11.80 |
| ADA | Adenosine Deaminase | Protein Coding | 11.05 |
| CEACAM3 | CEA Cell Adhesion Molecule 3 | Protein Coding | 11.05 |
| IL10 | Interleukin 10 | Protein Coding | 10.87 |
| CD40LG | CD40 Ligand | Protein Coding | 10.78 |
| SFTPA1 | Surfactant Protein A1 | Protein Coding | 10.66 |
| CXCL8 | C-X-C Motif Chemokine Ligand 8 | Protein Coding | 10.23 |
| MIR6758 | MicroRNA 6758 | RNA Gene | 10.13 |
| CSF1 | Colony Stimulating Factor 1 | Protein Coding | 10.12 |
| MUC1 | Mucin 1, Cell Surface Associated | Protein Coding | 10.00 |
| TNF | Tumor Necrosis Factor | Protein Coding | 9.67 |
| IL6 | Interleukin 6 | Protein Coding | 9.67 |
| ABCA1 | ATP Binding Cassette Subfamily A Member 1 | Protein Coding | 9.67 |
| NR1H3 | Nuclear Receptor Subfamily 1 Group H Member 3 | Protein Coding | 9.67 |
| IL3RA | Interleukin 3 Receptor Subunit Alpha | Protein Coding | 9.67 |
| USH2A | Usherin | Protein Coding | 8.84 |
| BMP1 | Bone Morphogenetic Protein 1 | Protein Coding | 6.40 |
| SAMD9L | Sterile Alpha Motif Domain Containing 9 Like | Protein Coding | 4.94 |
| DES | Desmin | Protein Coding | 4.53 |
| TERT | Telomerase Reverse Transcriptase | Protein Coding | 4.08 |
| CERNA3 | Competing Endogenous LncRNA 3 For MiR-645 | RNA Gene | 3.65 |
| FLT3 | Fms Related Receptor Tyrosine Kinase 3 | Protein Coding | 3.60 |
| STAT1 | Signal Transducer And Activator Of Transcription 1 | Protein Coding | 3.60 |
| MCM4 | Minichromosome Maintenance Complex Component 4 | Protein Coding | 3.60 |
| CEBPA | CCAAT Enhancer Binding Protein Alpha | Protein Coding | 3.60 |
| GATA1 | GATA Binding Protein 1 | Protein Coding | 3.60 |
| IRF8 | Interferon Regulatory Factor 8 | Protein Coding | 3.60 |
| STAG2 | STAG2 Cohesin Complex Component | Protein Coding | 3.60 |
| DOCK8 | Dedicator Of Cytokinesis 8 | Protein Coding | 3.60 |
| IFNGR2 | Interferon Gamma Receptor 2 | Protein Coding | 3.60 |
| IL12RB1 | Interleukin 12 Receptor Subunit Beta 1 | Protein Coding | 3.60 |
| ASXL1 | ASXL Transcriptional Regulator 1 | Protein Coding | 3.60 |
| DDX41 | DEAD-Box Helicase 41 | Protein Coding | 3.60 |
| U2AF1 | U2 Small Nuclear RNA Auxiliary Factor 1 | Protein Coding | 3.60 |
| GINS1 | GINS Complex Subunit 1 | Protein Coding | 3.60 |
| SETBP1 | SET Binding Protein 1 | Protein Coding | 3.60 |
| SRP72 | Signal Recognition Particle 72 | Protein Coding | 3.60 |
| ANKRD26 | Ankyrin Repeat Domain Containing 26 | Protein Coding | 3.60 |
| LUC7L2 | LUC7 Like 2, Pre-MRNA Splicing Factor | Protein Coding | 3.60 |
| SAMD9 | Sterile Alpha Motif Domain Containing 9 | Protein Coding | 3.60 |
| ZNF274 | Zinc Finger Protein 274 | Protein Coding | 3.60 |
| YIPF2 | Yip1 Domain Family Member 2 | Protein Coding | 3.60 |
| VDR | Vitamin D Receptor | Protein Coding | 3.33 |
| CYP27A1 | Cytochrome P450 Family 27 Subfamily A Member 1 | Protein Coding | 3.33 |
| CHI3L1 | Chitinase 3 Like 1 | Protein Coding | 3.33 |
| CXCR1 | C-X-C Motif Chemokine Receptor 1 | Protein Coding | 3.33 |
| SOAT1 | Sterol O-Acyltransferase 1 | Protein Coding | 3.33 |
| CCL7 | C-C Motif Chemokine Ligand 7 | Protein Coding | 3.33 |
| CCL4 | C-C Motif Chemokine Ligand 4 | Protein Coding | 3.33 |
| CCL8 | C-C Motif Chemokine Ligand 8 | Protein Coding | 3.33 |
| DEFB1 | Defensin Beta 1 | Protein Coding | 3.33 |
| CRLF2 | Cytokine Receptor Like Factor 2 | Protein Coding | 3.33 |
| NEAT1 | Nuclear Paraspeckle Assembly Transcript 1 | RNA Gene | 3.33 |
| MALAT1 | Metastasis Associated Lung Adenocarcinoma Transcript 1 | RNA Gene | 3.33 |
| PICSAR | P38 Inhibited Cutaneous Squamous Cell Carcinoma Associated LincRNA | RNA Gene | 3.33 |
| SNHG6 | Small Nucleolar RNA Host Gene 6 | RNA Gene | 3.33 |
| PWAR1 | Prader Willi/Angelman Region RNA 1 | RNA Gene | 3.33 |
| PTCSC2 | Papillary Thyroid Carcinoma Susceptibility Candidate 2 | RNA Gene | 3.33 |
| LOC112268276 | Uncharacterized LOC112268276 | RNA Gene | 3.33 |
| MIR3690 | MicroRNA 3690 | RNA Gene | 3.33 |
| ENSG00000228274 | Novel Transcript, Antisense To CBY1 | RNA Gene | 3.33 |
| XK | X-Linked Kx Blood Group Antigen, Kell And VPS13A Binding Protein | Protein Coding | 2.68 |
| HLA-DRB5 | Major Histocompatibility Complex, Class II, DR Beta 5 | Protein Coding | 2.68 |
| INTS12 | Integrator Complex Subunit 12 | Protein Coding | 2.68 |
| HLA-DRB9 | Major Histocompatibility Complex, Class II, DR Beta 9 (Pseudogene) | Pseudogene | 2.68 |
| MSH6 | MutS Homolog 6 | Protein Coding | 2.37 |
| ALMS1 | ALMS1 Centrosome And Basal Body Associated Protein | Protein Coding | 2.37 |
| IKBKG | Inhibitor Of Nuclear Factor Kappa B Kinase Regulatory Subunit Gamma | Protein Coding | 2.33 |
| CTSH | Cathepsin H | Protein Coding | 2.17 |
| CST3 | Cystatin C | Protein Coding | 2.17 |
| NAPSA | Napsin A Aspartic Peptidase | Protein Coding | 2.17 |
| ASS1 | Argininosuccinate Synthase 1 | Protein Coding | 1.93 |
| SLC3A1 | Solute Carrier Family 3 Member 1 | Protein Coding | 1.93 |
| OTC | Ornithine Transcarbamylase | Protein Coding | 1.93 |
| ASL | Argininosuccinate Lyase | Protein Coding | 1.93 |
| PRODH | Proline Dehydrogenase 1 | Protein Coding | 1.93 |
| SLC7A11 | Solute Carrier Family 7 Member 11 | Protein Coding | 1.93 |
| SLC3A2 | Solute Carrier Family 3 Member 2 | Protein Coding | 1.93 |
| SLC7A5 | Solute Carrier Family 7 Member 5 | Protein Coding | 1.93 |
| SLC1A7 | Solute Carrier Family 1 Member 7 | Protein Coding | 1.93 |
| SLC7A6 | Solute Carrier Family 7 Member 6 | Protein Coding | 1.93 |
| SLC7A8 | Solute Carrier Family 7 Member 8 | Protein Coding | 1.93 |
| OXA1L | OXA1L Mitochondrial Inner Membrane Protein | Protein Coding | 1.93 |
| TRA | T Cell Receptor Alpha Locus | Protein Coding | 1.93 |
| AIRE | Autoimmune Regulator | Protein Coding | 1.91 |
| IL3 | Interleukin 3 | Protein Coding | 1.90 |
| DMBT1 | Deleted In Malignant Brain Tumors 1 | Protein Coding | 1.57 |
| PPARG | Peroxisome Proliferator Activated Receptor Gamma | Protein Coding | 1.53 |
| CSF3R | Colony Stimulating Factor 3 Receptor | Protein Coding | 1.35 |
| IL5RA | Interleukin 5 Receptor Subunit Alpha | Protein Coding | 1.35 |
| MBL2 | Mannose Binding Lectin 2 | Protein Coding | 1.35 |
| CDH13 | Cadherin 13 | Protein Coding | 1.35 |
| CLEC12A | C-Type Lectin Domain Family 12 Member A | Protein Coding | 1.35 |
| IGHG1 | Immunoglobulin Heavy Constant Gamma 1 (G1m Marker) | Protein Coding | 1.35 |
| IGLC2 | Immunoglobulin Lambda Constant 2 | Protein Coding | 1.35 |
| ELANE | Elastase, Neutrophil Expressed | Protein Coding | 0.90 |
| NKX2-1 | NK2 Homeobox 1 | Protein Coding | 0.90 |
| PRTN3 | Proteinase 3 | Protein Coding | 0.90 |
| CTSG | Cathepsin G | Protein Coding | 0.90 |
| SCGB1A1 | Secretoglobin Family 1A Member 1 | Protein Coding | 0.90 |
| CSF3 | Colony Stimulating Factor 3 | Protein Coding | 0.90 |
| MRC1 | Mannose Receptor C-Type 1 | Protein Coding | 0.90 |
| SFTPA2 | Surfactant Protein A2 | Protein Coding | 0.90 |
| SFTA3 | Surfactant Associated 3 | RNA Gene | 0.90 |
| LINC02605 | Long Intergenic Non-Protein Coding RNA 2605 | RNA Gene | 0.90 |
| PGR-AS1 | PGR Antisense RNA 1 | RNA Gene | 0.90 |
| LOC117038771 | CRISPRi-FlowFISH-Validated H1-10 Regulatory Element 2 | Functional Element | 0.77 |
| IL5 | Interleukin 5 | Protein Coding | 0.63 |
| CALR | Calreticulin | Protein Coding | 0.44 |
| RUNX1 | RUNX Family Transcription Factor 1 | Protein Coding | 0.44 |
| SLC11A2 | Solute Carrier Family 11 Member 2 | Protein Coding | 0.44 |
| SLC40A1 | Solute Carrier Family 40 Member 1 | Protein Coding | 0.44 |
| ETFDH | Electron Transfer Flavoprotein Dehydrogenase | Protein Coding | 0.44 |
| MT-CYB | Mitochondrially Encoded Cytochrome B | Protein Coding | 0.44 |
| LINC00472 | Long Intergenic Non-Protein Coding RNA 472 | RNA Gene | 0.44 |
| LINC00336 | Long Intergenic Non-Protein Coding RNA 336 | RNA Gene | 0.44 |
| ZFAS1 | ZNFX1 Antisense RNA 1 | RNA Gene | 0.44 |
| MIR6852 | MicroRNA 6852 | RNA Gene | 0.44 |

1. The Relevance score, sourced from the GeneCards database (https://www.genecards.org/), indicates the degree of relevance of each gene to the research topic. This score is calculated by considering a variety of factors, including the frequency of the gene's appearance in related research literature, known associations with specific diseases or conditions, and other relevant bioinformatics parameters. A high Relevance score suggests a strong relevance of the gene to the research topic.
