# Supplementary material for: Phenotypic heterogeneity in mortality and prognosis of pulmonary alveolar proteinosis: a large-scale, global pooled analysis of individual-level data
Source: Orphanet J Rare Dis. 2025 Mar 4;20:102. doi: 10.1186/s13023-025-03617-3 (PMC11881271; doi:10.1186/s13023-025-03617-3)
Supplement: Supplementary file 10 — Supplementary Material 10.Table A10: Summary of genes and descriptions related to Autoimmune PAP in GeneCards Database. [file 13023_2025_3617_MOESM10_ESM.docx]

**Table A10** Summary of genes and descriptions related to Autoimmune PAP in GeneCards Database.

| Gene Symbol | Description | Category | Relevance score |
| --- | --- | --- | --- |
| HLA-DRB1 | Major Histocompatibility Complex, Class II, DR Beta 1 | Protein Coding | 20.29 |
| CSF2 | Colony Stimulating Factor 2 | Protein Coding | 4.34 |
| NEAT1 | Nuclear Paraspeckle Assembly Transcript 1 | RNA Gene | 3.07 |
| MALAT1 | Metastasis Associated Lung Adenocarcinoma Transcript 1 | RNA Gene | 3.07 |
| PICSAR | P38 Inhibited Cutaneous Squamous Cell Carcinoma Associated LincRNA | RNA Gene | 3.07 |
| SNHG6 | Small Nucleolar RNA Host Gene 6 | RNA Gene | 3.07 |
| PTCSC2 | Papillary Thyroid Carcinoma Susceptibility Candidate 2 | RNA Gene | 3.07 |
| LOC112268276 | Uncharacterized LOC112268276 | RNA Gene | 3.07 |
| ENSG00000228274 | Novel Transcript, Antisense To CBY1 | RNA Gene | 3.07 |
| CSF2RB | Colony Stimulating Factor 2 Receptor Subunit Beta | Protein Coding | 2.52 |

1. The Relevance score, sourced from the GeneCards database (https://www.genecards.org/), indicates the degree of relevance of each gene to the research topic. This score is calculated by considering a variety of factors, including the frequency of the gene's appearance in related research literature, known associations with specific diseases or conditions, and other relevant bioinformatics parameters. A high Relevance score suggests a strong relevance of the gene to the research topic.
