# Supplementary material for: Phenotypic heterogeneity in mortality and prognosis of pulmonary alveolar proteinosis: a large-scale, global pooled analysis of individual-level data
Source: Orphanet J Rare Dis. 2025 Mar 4;20:102. doi: 10.1186/s13023-025-03617-3 (PMC11881271; doi:10.1186/s13023-025-03617-3)
Supplement: Supplementary file 11 — Supplementary Material 11.Table A11: Summary of genes and descriptions related to Secondary PAP in GeneCards Database. [file 13023_2025_3617_MOESM11_ESM.docx]

**Table A11** Summary of genes and descriptions related to Secondary PAP in GeneCards Database.

| Gene Symbol | Description | Category | Relevance score |
| --- | --- | --- | --- |
| HLA-DRB1 | Major Histocompatibility Complex, Class II, DR Beta 1 | Protein Coding | 7.94 |
| CDH13 | Cadherin 13 | Protein Coding | 0.54 |
| SFTPD | Surfactant Protein D | Protein Coding | 0.54 |

1. The Relevance score, sourced from the GeneCards database (https://www.genecards.org/), indicates the degree of relevance of each gene to the research topic. This score is calculated by considering a variety of factors, including the frequency of the gene's appearance in related research literature, known associations with specific diseases or conditions, and other relevant bioinformatics parameters. A high Relevance score suggests a strong relevance of the gene to the research topic.
