# Supplementary material for: Phenotypic heterogeneity in mortality and prognosis of pulmonary alveolar proteinosis: a large-scale, global pooled analysis of individual-level data
Source: Orphanet J Rare Dis. 2025 Mar 4;20:102. doi: 10.1186/s13023-025-03617-3 (PMC11881271; doi:10.1186/s13023-025-03617-3)
Supplement: Supplementary file 12 — Supplementary Material 12.Table A12: Summary of genes and descriptions related to Hereditary PAP in GeneCards Database. [file 13023_2025_3617_MOESM12_ESM.docx]

**Table A12** Summary of genes and descriptions related to Hereditary PAP in GeneCards Database.

| Gene Symbol | Description | Category | Relevance score |
| --- | --- | --- | --- |
| ABCA3 | ATP Binding Cassette Subfamily A Member 3 | Protein Coding | 111.60 |
| SFTPB | Surfactant Protein B | Protein Coding | 64.21 |
| SFTPC | Surfactant Protein C | Protein Coding | 56.50 |
| CSF2RB | Colony Stimulating Factor 2 Receptor Subunit Beta | Protein Coding | 26.09 |
| CSF2RA | Colony Stimulating Factor 2 Receptor Subunit Alpha | Protein Coding | 25.51 |
| BMP1 | Bone Morphogenetic Protein 1 | Protein Coding | 7.59 |
| FARSB | Phenylalanyl-TRNA Synthetase Subunit Beta | Protein Coding | 7.41 |
| MARS1 | Methionyl-TRNA Synthetase 1 | Protein Coding | 7.41 |
| IL3RA | Interleukin 3 Receptor Subunit Alpha | Protein Coding | 2.51 |
| CRLF2 | Cytokine Receptor Like Factor 2 | Protein Coding | 2.51 |

1. The Relevance score, sourced from the GeneCards database (https://www.genecards.org/), indicates the degree of relevance of each gene to the research topic. This score is calculated by considering a variety of factors, including the frequency of the gene's appearance in related research literature, known associations with specific diseases or conditions, and other relevant bioinformatics parameters. A high Relevance score suggests a strong relevance of the gene to the research topic.
