# Supplementary figures and images for: Phenotypic heterogeneity in mortality and prognosis of pulmonary alveolar proteinosis: a large-scale, global pooled analysis of individual-level data
Source: Orphanet J Rare Dis. 2025 Mar 4;20:102. doi: 10.1186/s13023-025-03617-3 (PMC11881271; doi:10.1186/s13023-025-03617-3)

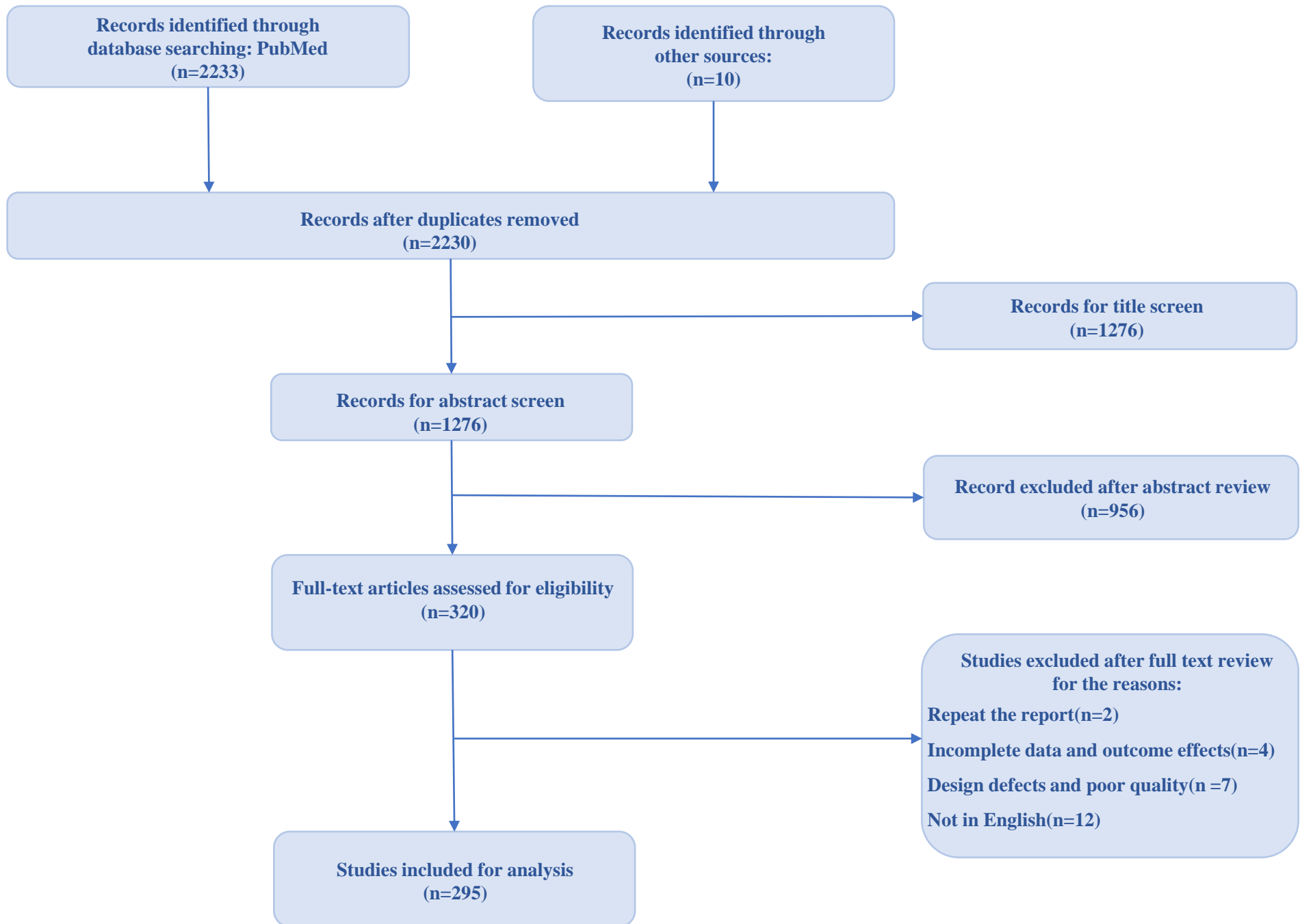

Supplement: Supplementary file 13 — Supplementary Material 13: Figure S1. Flow chart of studies included in the Global PAP Pooled Analysis. [file 13023_2025_3617_MOESM13_ESM.pdf]

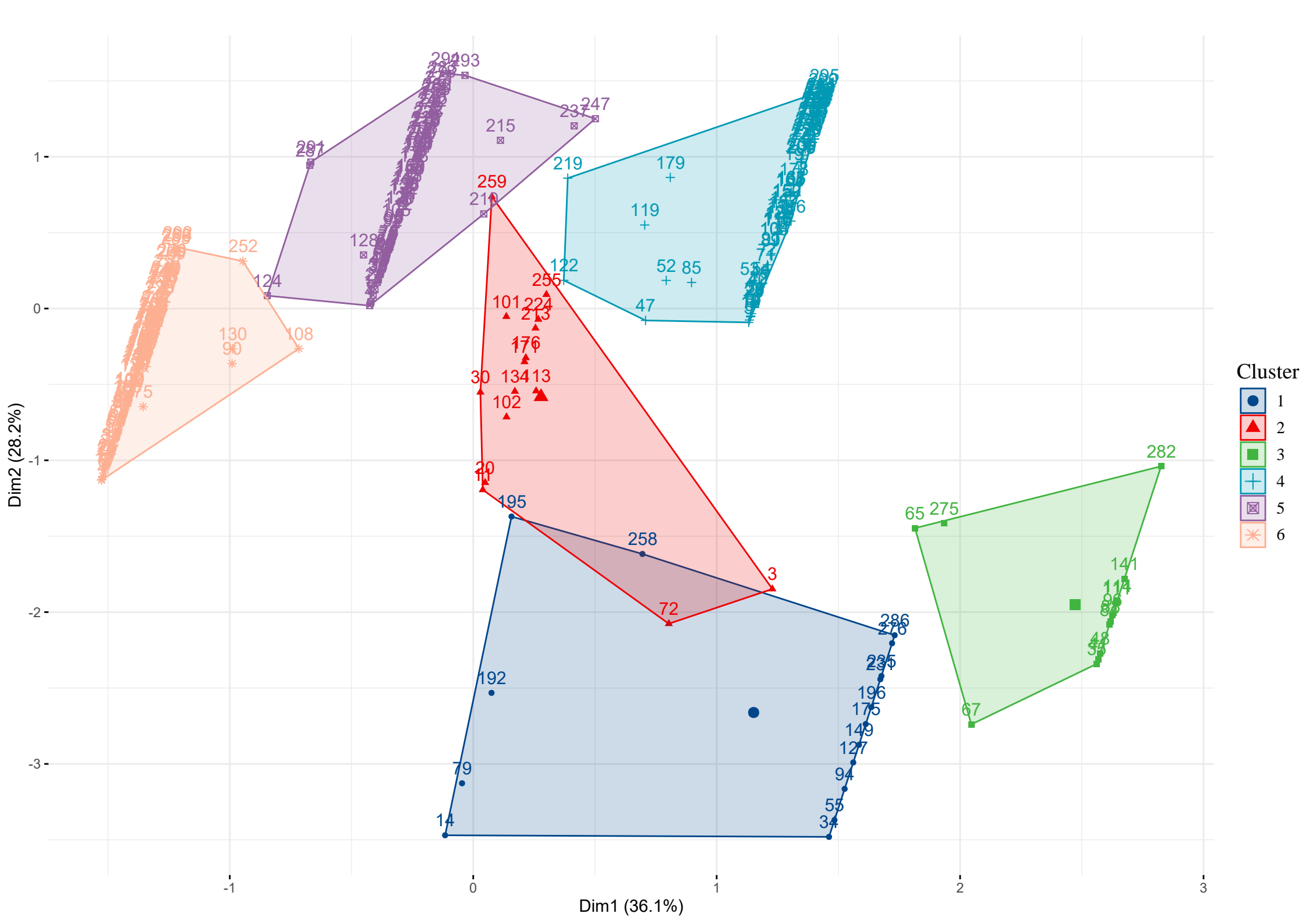

Supplement: Supplementary file 14 — Supplementary Material 14: Figure S2. Cluster distribution map of Kmeans algorithm based on three respiratory symptoms. [file 13023_2025_3617_MOESM14_ESM.pdf]

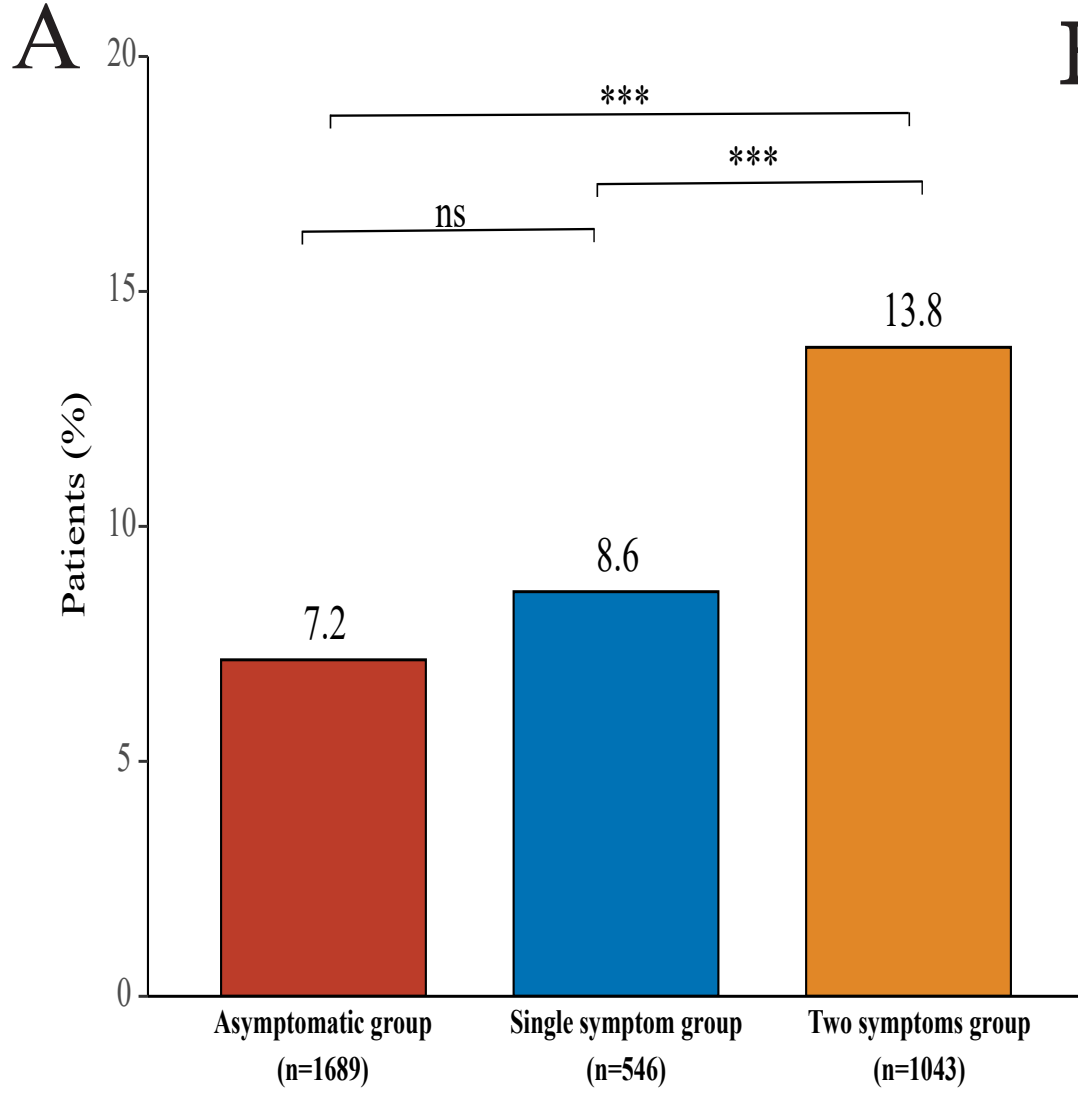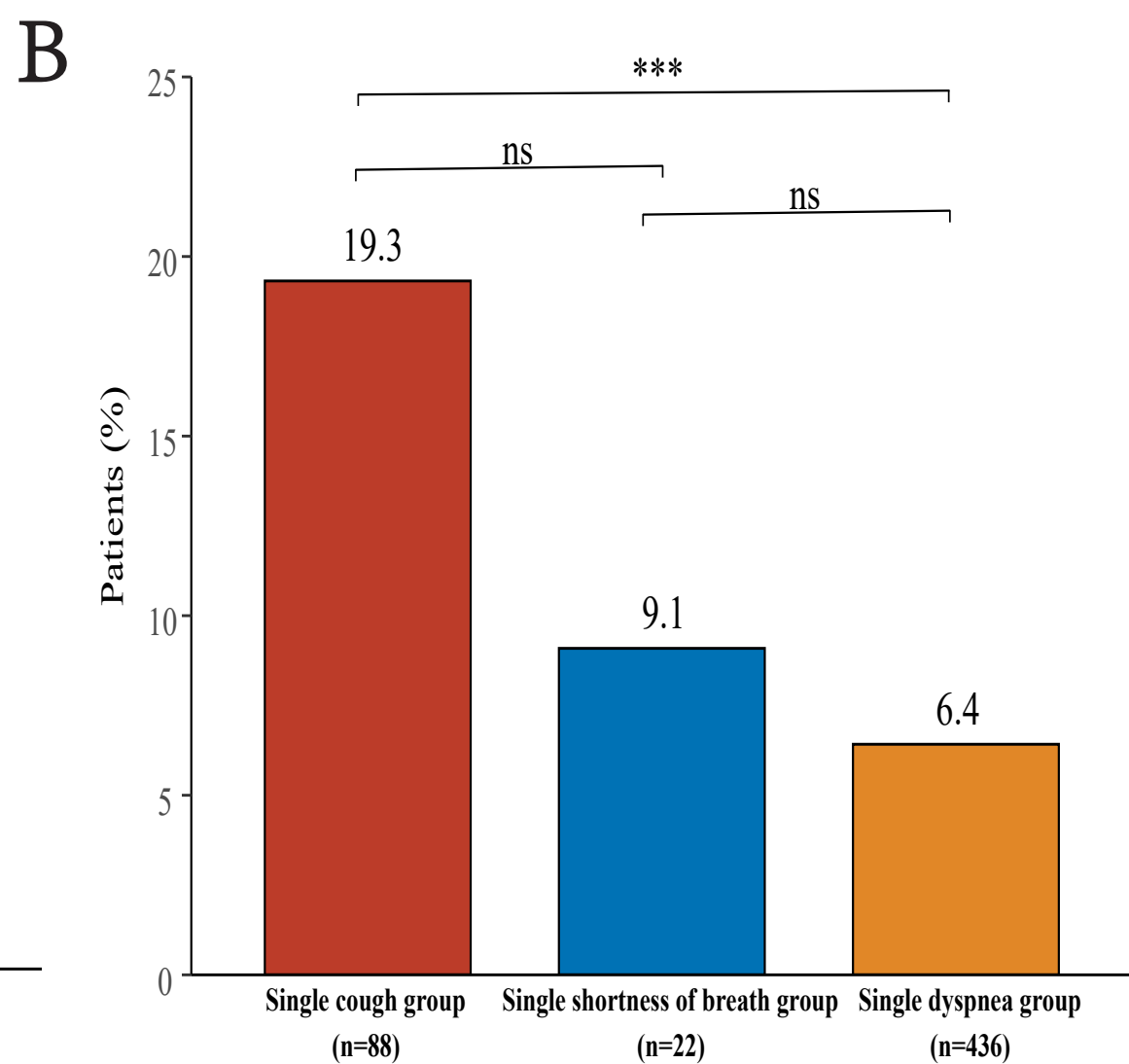

Supplement: Supplementary file 15 — Supplementary Material 15: Figure S3. Histogram of mortality distribution on clustering results. (A)Histogram of mortality distribution for three types based on clustering results; (B) Histogram of mortality distribution in a single symptom group based on clustering results. [file 13023_2025_3617_MOESM15_ESM.pdf]

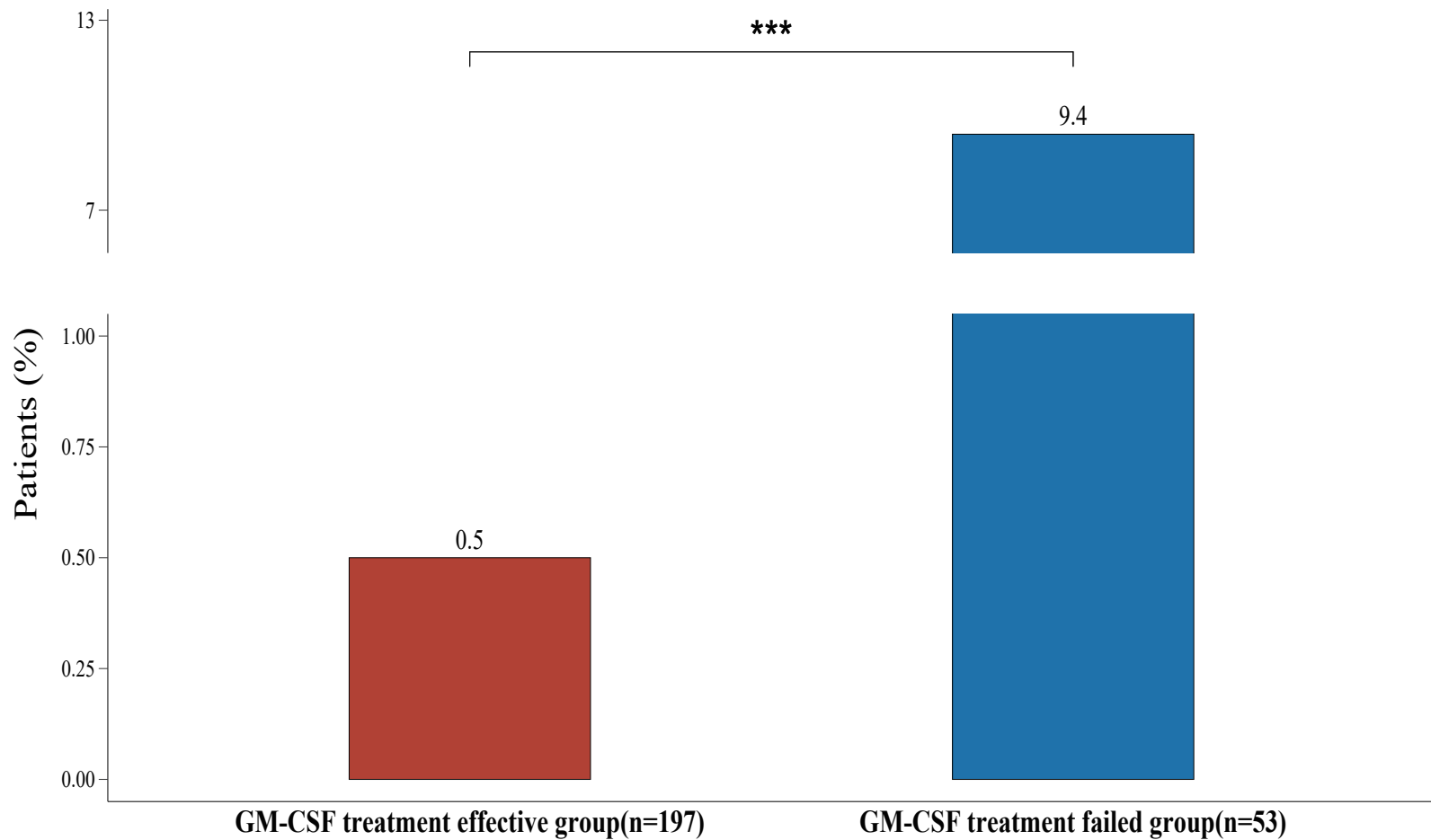

Supplement: Supplementary file 16 — Supplementary Material 16: Figure S4. Effect of GM-CSF treatment effectiveness on mortality in PAP population. Abbreviations: GM-CSF, granulocyte-macrophage colony-stimulating factor. [file 13023_2025_3617_MOESM16_ESM.pdf]

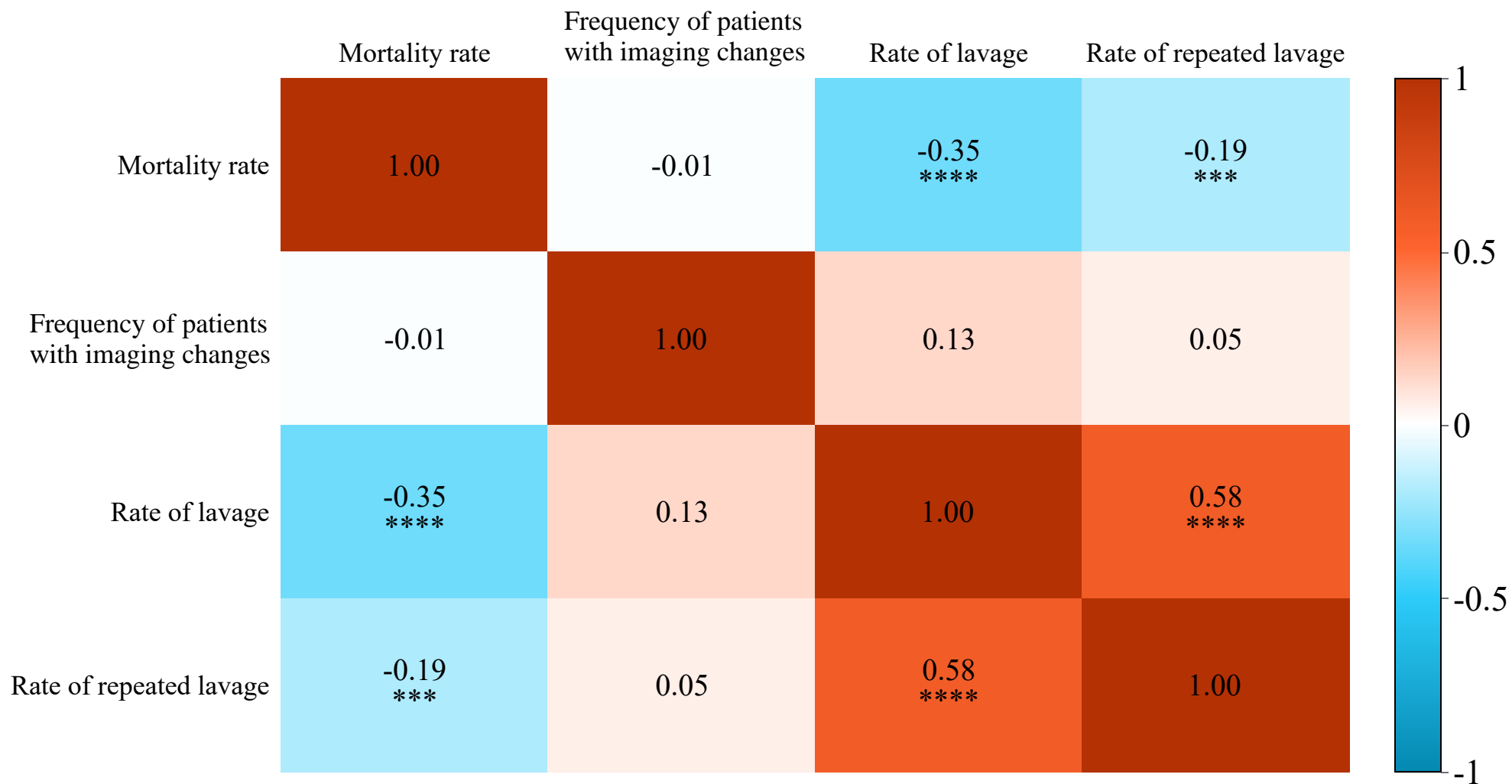

Supplement: Supplementary file 17 — Supplementary Material 17: Figure S5. Heat map of correlation between lavage rate, CT typical lesion rate and cohort mortality. [file 13023_2025_3617_MOESM17_ESM.pdf]

**A**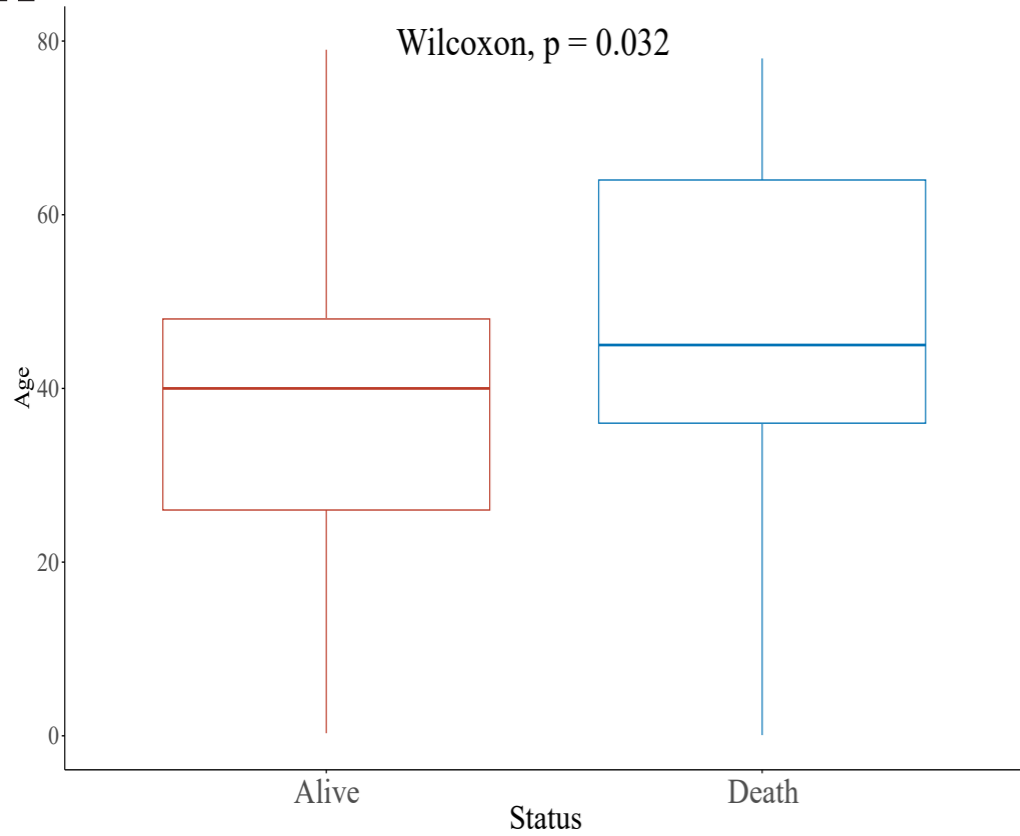**B**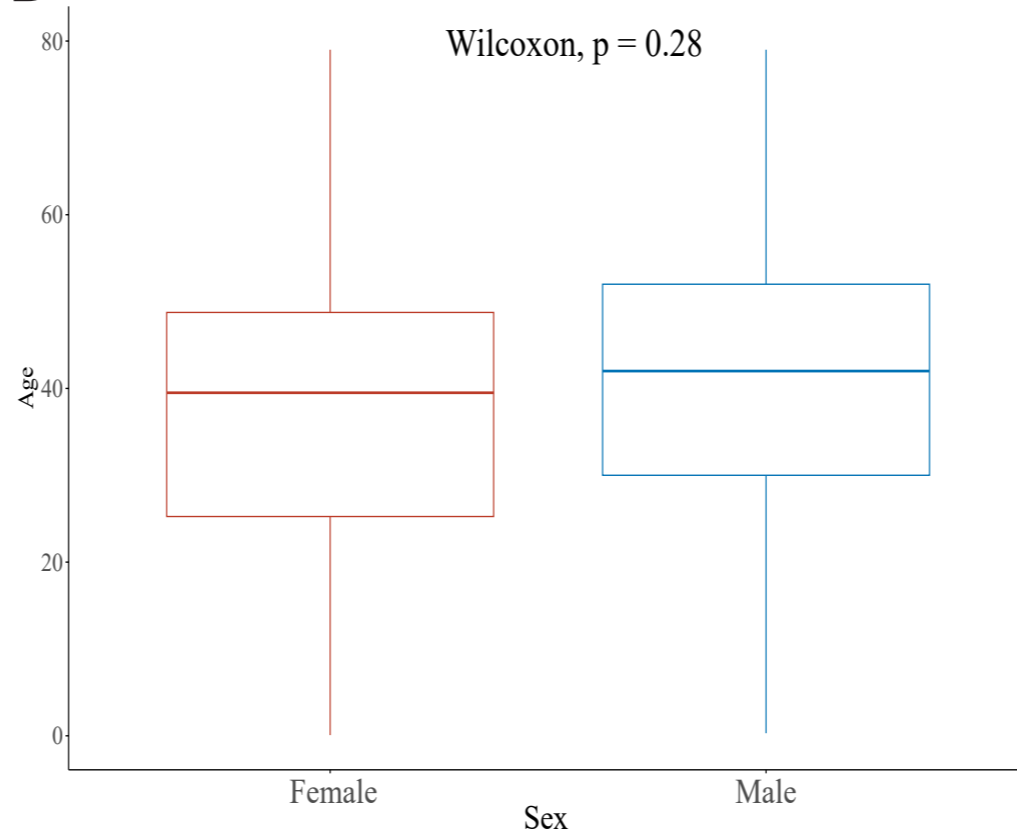**C**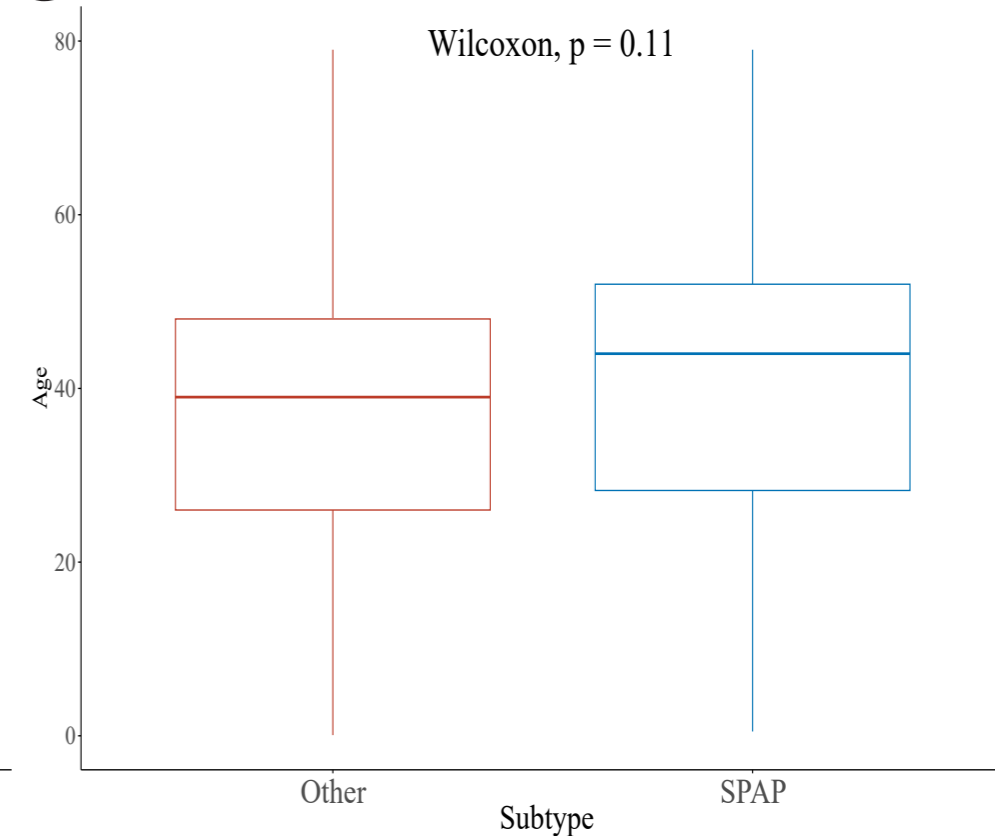

Supplement: Supplementary file 19 — Supplementary Material 19: Figure S7. Comparison of different groups in terms of age. (A)Comparison of age distribution of clinical outcomes; (B) Comparison of age distribution of sex; (C) Comparison of age distribution of disease type. [file 13023_2025_3617_MOESM19_ESM.pdf]

A

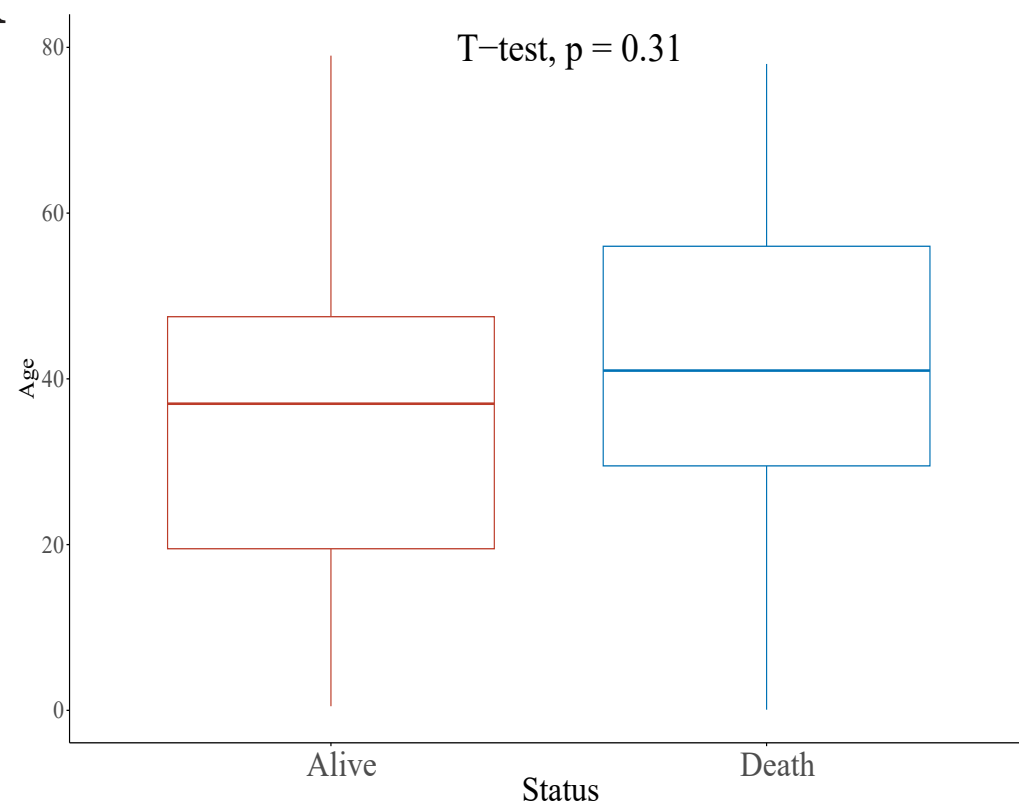

B

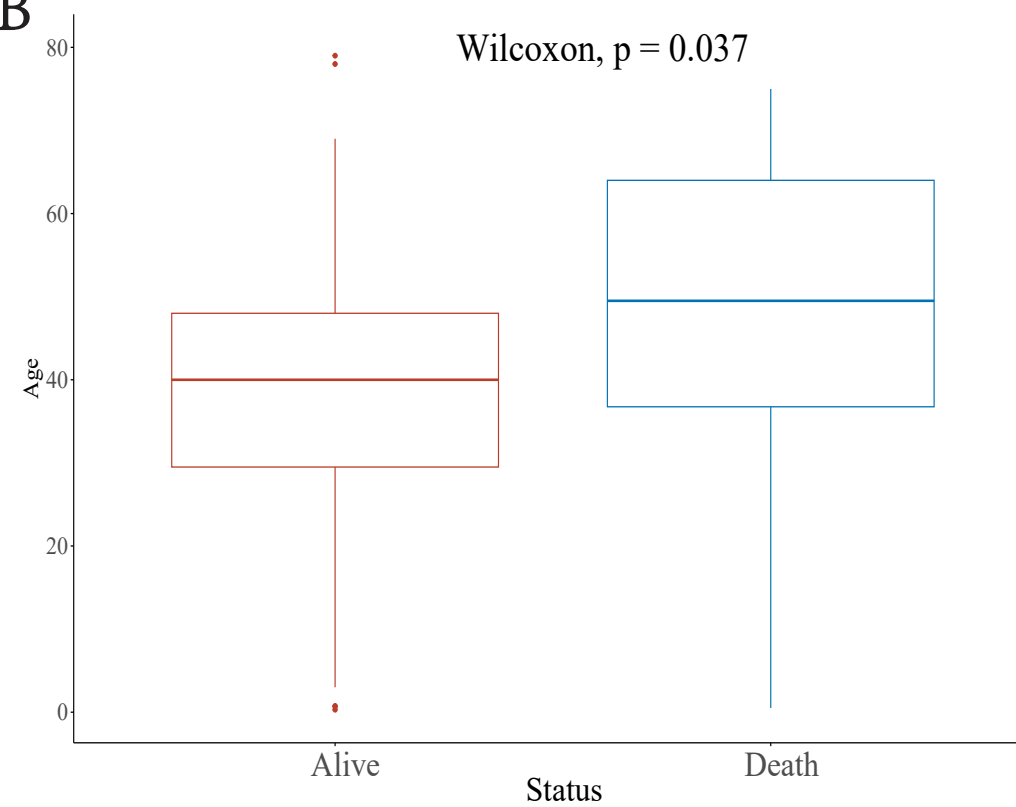

C

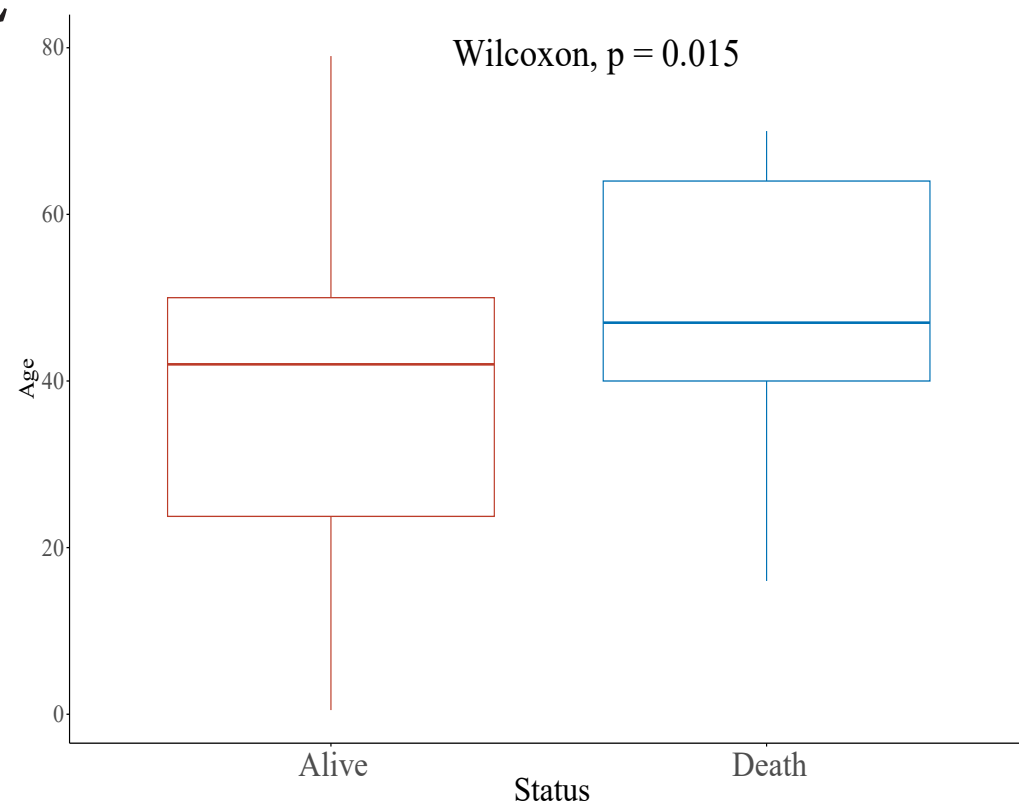

D

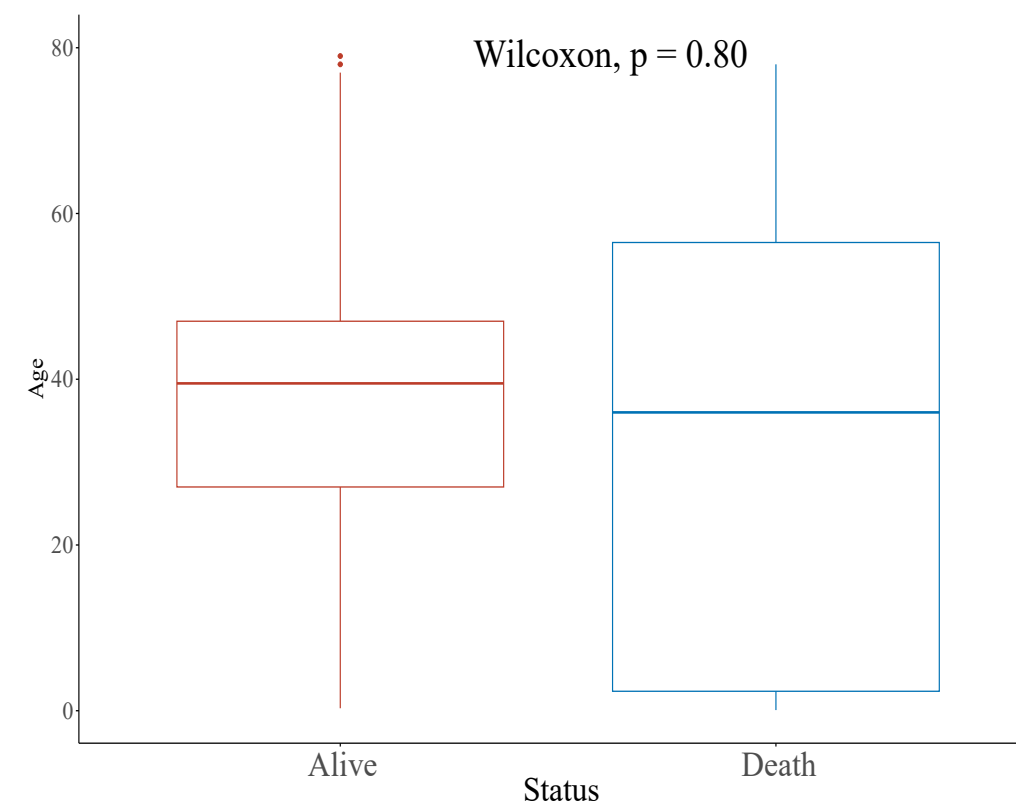

Supplement: Supplementary file 20 — Supplementary Material 20: Figure S8. Comparison of age distribution in survival status between different subgroups. (A) Comparison of age distribution of clinical outcomes in female subgroup; (B) Comparison of age distribution of clinical outcomes in male subgroup; (C) Comparison of age distribution of clinical outcomes in SPAP subgroup; (D) Comparison of age distribution of clinical outcomes in other type of PAP subgroup. [file 13023_2025_3617_MOESM20_ESM.pdf]
