# Supplementary material for: Phenotypic heterogeneity in mortality and prognosis of pulmonary alveolar proteinosis: a large-scale, global pooled analysis of individual-level data
Source: Orphanet J Rare Dis. 2025 Mar 4;20:102. doi: 10.1186/s13023-025-03617-3 (PMC11881271; doi:10.1186/s13023-025-03617-3)
Supplement: Supplementary file 18 — Supplementary Material 18: Figure S6. Distribution of 211 PAP patients in individual analysis. (A)Distribution of 211 PAP patients by sex and disease type; (B) Age distribution of 211 PAP patients; (C) Age distribution of clinical outcomes grouped by sex; (D) Age distribution of clinical outcomes grouped by disease type. Abbreviations: PAP, pulmonary alveolar proteinosis; SPAP: secondary pulmonary alveolar proteinosis. [file 13023_2025_3617_MOESM18_ESM.pdf]

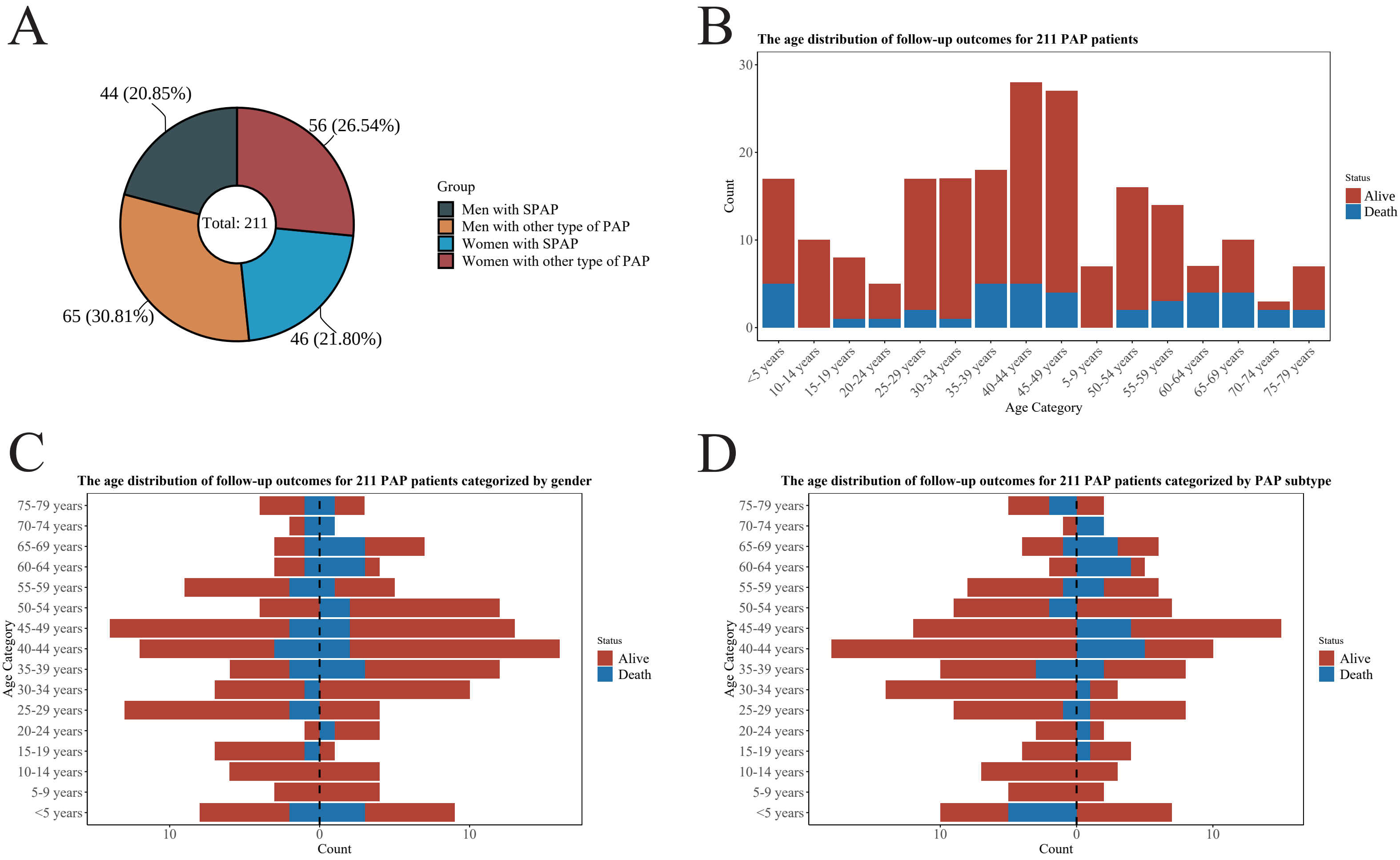
A
44 (20.85%)
56 (26.54%)
65 (30.81%)
46 (21.80%)
Total: 211
Group
Men with SPAP
Men with other type of PAP
Women with SPAP
Women with other type of PAP
B
The age distribution of follow-up outcomes for 211 PAP patients
Count
Age Category
<5 years
10-14 years
15-19 years
20-24 years
25-29 years
30-34 years
35-39 years
40-44 years
45-49 years
5-9 years
50-54 years
55-59 years
60-64 years
65-69 years
70-74 years
75-79 years
Status
Alive
Death
C
The age distribution of follow-up outcomes for 211 PAP patients categorized by gender
Age Category
Count
Status
Alive
Death
D
The age distribution of follow-up outcomes for 211 PAP patients categorized by PAP subtype
Age Category
Count
Status
Alive
Death
