# Supplementary material for: Phenotypic heterogeneity in mortality and prognosis of pulmonary alveolar proteinosis: a large-scale, global pooled analysis of individual-level data
Source: Orphanet J Rare Dis. 2025 Mar 4;20:102. doi: 10.1186/s13023-025-03617-3 (PMC11881271; doi:10.1186/s13023-025-03617-3)
Supplement: Supplementary file 21 — Supplementary Material 21.Detailed methodological information and description. [file 13023_2025_3617_MOESM21_ESM.docx]

**Supplement Material**

**Search strategy and selection criteria**

A systematic literature search was conducted in PubMed between 1,950 and 2,021, using the following keywords: “Pulmonary alveolar proteinosis” and “Alveolar proteinosis” or both alone to identify articles. This search was restricted to human studies in the English language. Eligible studies were included for a pooled analysis if they met the following inclusion criteria: (1) studies that included patients with a pathologically confirmed diagnosis of PAP; (2) studies that reported relevant clinical data of patients with PAP. Exclusion criteria were as follows: (1) studies that was repeated report; (2) study data were incomplete or there were outcome effects; (3) study design was flawed or of poor quality; (4) studies are published in non-English. A flowchart regarding study inclusion can be found in **Supplement Figure A1**.

**Population Inclusion and Clinical Characteristics**

PAP was defined as a diagnosis established by histopathologic examination of material from open lung biopsies, transbronchial biopsies, or bronchoalveolar lavage. Concerning different classifications, idiopathic PAP (IPAP) is defined as PAP types observed in patients over the age of 18, with unclear etiology or those related to GM-CSF mechanisms. Secondary PAP (SPAP) is associated with other types of diseases, such as leukemia or genetic immune deficiencies. Congenital PAP (CPAP) is characterized by diagnosis before the age of 18 or the presence of specific genetic mutations related to surfactant metabolism. Specifically, we extracted and analyzed the following data from individual case reports or series: demographic and clinical features (age, sex, symptoms, etc.), diagnostic methods, and treatment interventions. Additionally, Clinical phenotype characteristics and frequency of occurrence related to PAP were accessed from the Orphanet (https://www.orphadata.com/) and the Human Phenotype Ontology database (https://hpo.jax.org/app/), aiming to supplement our aggregated clinical features. Furthermore, to clarify the comorbidities in IPAP and the characteristics of secondary factors in SPAP, the comorbidities in IPAP patients and the characteristics of secondary factors in SPAP patients were summarized. Regarding CPAP, Mutation-related genes in CPAP were collated from some studies. Genes related to PAP and its various types were reported based on past research findings from the GeneCards database(https://www.genecards.org/).

**Prognostic Outcomes**

Clinical outcomes in PAP patients during follow-up were defined as: 1) Spontaneous remission: gradual reduction or disappearance of symptoms and clinical manifestations without specific therapeutic intervention. 2) Stable: symptoms and clinical manifestations do not change significantly with or without intervention. 3) Death: patients died from various causes during the follow-up period. In the exploration of mortality-related factors in the total PAP population, attention was given to three common clinical symptoms of PAP: coughing, breathing difficulties, and shortness of breath. The incidence of each symptom in the studies included was calculated and clustered, aiming to investigate the impact of varying symptom incidence rates on clinical characteristics and mortality. In addition, the relationship between therapeutic interventions and PAP typing and death was also explored. The therapeutic effect of GM-CSF was divided into effective treatment and ineffective treatment according to the treatment status reported in the study. Furthermore, data from individualized patients with clear clinical information and follow-up outcomes were included. The analysis of mortality-related factors in small sample cohorts was conducted to further validate findings applicable to the broader population.
